# Whole-Genome Sequencing of *Escherichia coli* O18ab:H11 from South African Beef: Antimicrobial Resistance, Virulence, and a Rare Sequence Type

**DOI:** 10.3390/microorganisms14092003

**Published:** 2026-09-09

**Authors:** Fezeka P. Ndlazi, Ephifania Geza, Evelyn Madoroba

**Affiliations:** 1 Department of Biochemistry and Microbiology, University of Zululand, Private Bag X1001, KwaDlangezwa, Empangeni 3886, South Africa; fezyndlazy@gmail.com; 2 Computational Biology Division, Faculty of Health Sciences, University of Cape Town, Observatory 7925, South Africa; ephie.geza@uct.ac.za or ephie@aims.ac.za

**Keywords:** *Escherichia coli* (*E. coli*), WGS, antimicrobial resistance, virulence genes, pan-genome analysis

## Abstract

Antimicrobial-resistant *Escherichia coli* in food systems represents a growing public health concern, yet whole-genome sequencing (WGS)-based surveillance of beef-associated *E. coli* in South Africa remains limited. This study applied WGS to comprehensively characterise the antimicrobial resistance (AMR) profile, virulence gene repertoire, sequence type, and serotype of an *E. coli* isolate recovered from retail beef in KwaZulu-Natal Province, South Africa, and to contextualise these findings within the broader African *E. coli* population through comparative genomic analysis of 397 publicly available *E. coli* genome sequences retrieved from the NCBI SRA. The isolate was recovered by classical microbiological methods, confirmed by MALDI-TOF MS, and whole-genome-sequenced on the PacBio Onso platform. In silico serotyping assigned the genome to serotype O18ab:H11. MLST under the Achtman seven-locus scheme assigned the genome to ST7106, a sequence type with no prior record in South Africa. The isolate carried the intrinsic β-lactamase gene *bla*_EC-15_ (reported as chromosomal in the literature), conferring narrow-spectrum resistance to aminopenicillins, alongside an acquired resistome comprising *aadA1*, *tet*(B), and *sat2*, conferring resistance to aminoglycosides, tetracyclines, and streptothricin; no ESBL or carbapenemase genes were detected. The virulence gene repertoire—encompassing type 1 fimbriae (*fimA–I*), curli fibres (*csgA–G*), the *E. coli* common pilus (*ecpRABCDE*), enterobactin iron-acquisition genes, *hlyE*, and the invasion genes *ibeB* and *ibeC*—indicates colonisation capacity, but *ibeB*, *ibeC*, and *hlyE* showed no clinical enrichment relative to carriage genomes, and the isolate showed no close genomic relationships with any clinical genome; a definitive extra-intestinal pathogenic *E. coli* (ExPEC) pathotype assignment is therefore not supported. PathogenFinder returned a pathogenicity probability of 0.944 (532 pathogen-associated protein families detected), without in vivo corroboration. Comparative analysis identified a pan-resistome of 97 unique AMR genes across the 398 genomes, including *bla*_CTX-M-15_ (59.0%) and *bla*_NDM-5_ (49.5%), predominantly among clinical genomes. Pan-genome analysis revealed an open genomic structure (α=0.194; 16,578 gene clusters), and core-genome phylogenetics confirmed the beef-derived isolate as a genetically distinct lineage (minimum pairwise SNP distance: 1629 SNPs). These findings demonstrate the presence of a genomically distinct, potentially pathogenic *E. coli* lineage in the South African beef supply and highlight the importance of integrating WGS into routine food safety and AMR surveillance frameworks across the region.

## 1. Introduction

*Escherichia coli* is a Gram-negative bacterium that colonises the gastro-intestinal tracts of humans and animals as a commensal organism [1]. Some strains, however, have acquired virulence factors that enable them to cause a broad spectrum of diseases, including urinary tract infections, neonatal meningitis, septicaemia, haemolytic–uraemic syndrome, and gastro-intestinal illness [2,3,4].

Pathogenic *E. coli* strains are broadly classified into intestinal and extra-intestinal pathotypes based on their virulence gene repertoire and associated disease syndromes. Intestinal pathotypes include enteropathogenic (EPEC), enterotoxigenic (ETEC), enteroinvasive (EIEC), diffusely adherent (DAEC), enteroaggregative (EAEC), and Shiga toxin-producing (STEC) *E. coli*, while extra-intestinal pathotypes include uropathogenic (UPEC), neonatal meningitis (NMEC), avian pathogenic (APEC), and sepsis-associated (SEPEC) *E. coli* [5,6,7]. These pathotypes are distinguished by distinct combinations of adhesins, toxins, iron-acquisition systems, and immune evasion factors that collectively determine their tropism and pathogenic potential [8].

The emergence and global dissemination of antimicrobial-resistant *E. coli* represent a critical public health threat. The WHO estimated that antimicrobial resistance (AMR) was directly responsible for 1.27 million deaths globally in 2019, with *E. coli* identified as one of the principal bacterial contributors [9,10]. Beyond its clinical significance, *E. coli* serves as a sentinel organism for monitoring resistance trends across human, animal, and environmental reservoirs, a role central to One Health surveillance frameworks [11]. The beef production chain is a particularly important transmission pathway, with antimicrobial-resistant *E. coli* documented at multiple points from farm to fork [12,13,14]. Resistance to ampicillin, tetracycline, and sulfonamides has been widely reported in beef-associated genomes [15,16,17,18]. At the genetic level, resistance arises through both intrinsic and acquired mechanisms. Intrinsic mechanisms include reduced outer membrane permeability and constitutive efflux pump expression, whereas acquired resistance is primarily mediated by horizontal gene transfer through conjugation, transformation, and transduction [19,20,21,22].

The public health significance of *E. coli* in Africa is considerable. In South Africa alone, 327 food-borne disease outbreaks between 2013 and 2017 resulted in 11,155 illnesses and 49 deaths [23], highlighting the consequences of inadequate food safety surveillance. Across the continent, diverse *E. coli* strains have been isolated from animals and food products [24]; however, the molecular epidemiology of these strains remains poorly characterised. In South Africa specifically, existing studies on beef-derived *E. coli* have largely relied on phenotypic and targeted molecular approaches [25,26], while whole-genome sequencing (WGS)-based characterisation remains notably absent from the literature. This gap is particularly important given that the co-occurrence of AMR determinants and virulence factors in meat-associated *E. coli* underscores the urgent need for genomic surveillance to support risk assessment and guide intervention strategies [27].

The studies on *E. coli* from beef and beef-based products in South Africa present some conceptual gaps regarding genomic baseline data and One Health transmission comparison of antimicrobial resistance factors and other molecular epidemiological characteristics. Some existing studies focus on localised prevalence [28,29,30] rather than detailed whole-genome sequencing (WGS)-based surveillance. The whole-genome sequencing data for *E. coli* in South Africa is limited [31,32,33], usually leaving beef-associated resistomes under-characterised. One Health studies tracking *E. coli* recovered along the beef value chain are crucial because they map movement of AMR across diverse ecosystems. There is a gap in geographic representation whereby rural provinces such as KwaZulu-Natal have historically been underrepresented in WGS-based surveillance of *E. coli* recovered from beef and One Health studies. In addition, *E. coli* studies from beef focused on STEC O157:H7, and non-O157 serogroup isolation and characterisation [27], leaving distinct literature gaps on other *E. coli* pathotypes.

Addressing these gaps requires WGS-based surveillance, which enables simultaneous characterisation of AMR determinants, virulence gene repertoires, sequence types (STs), serotypes, and phylogenetic relationships at a resolution unattainable by conventional typing methods [34]. This study therefore aimed to comprehensively characterise an *E. coli* isolate recovered from retail beef in KwaZulu-Natal, South Africa, using WGS. The specific objectives were to determine its ST, serotype, AMR profile, and virulence gene content and to contextualise these findings within the broader African *E. coli* population through comparative genomic analysis.

## 2. Materials and Methods

### 2.1. Study Design

**Figure 1:**
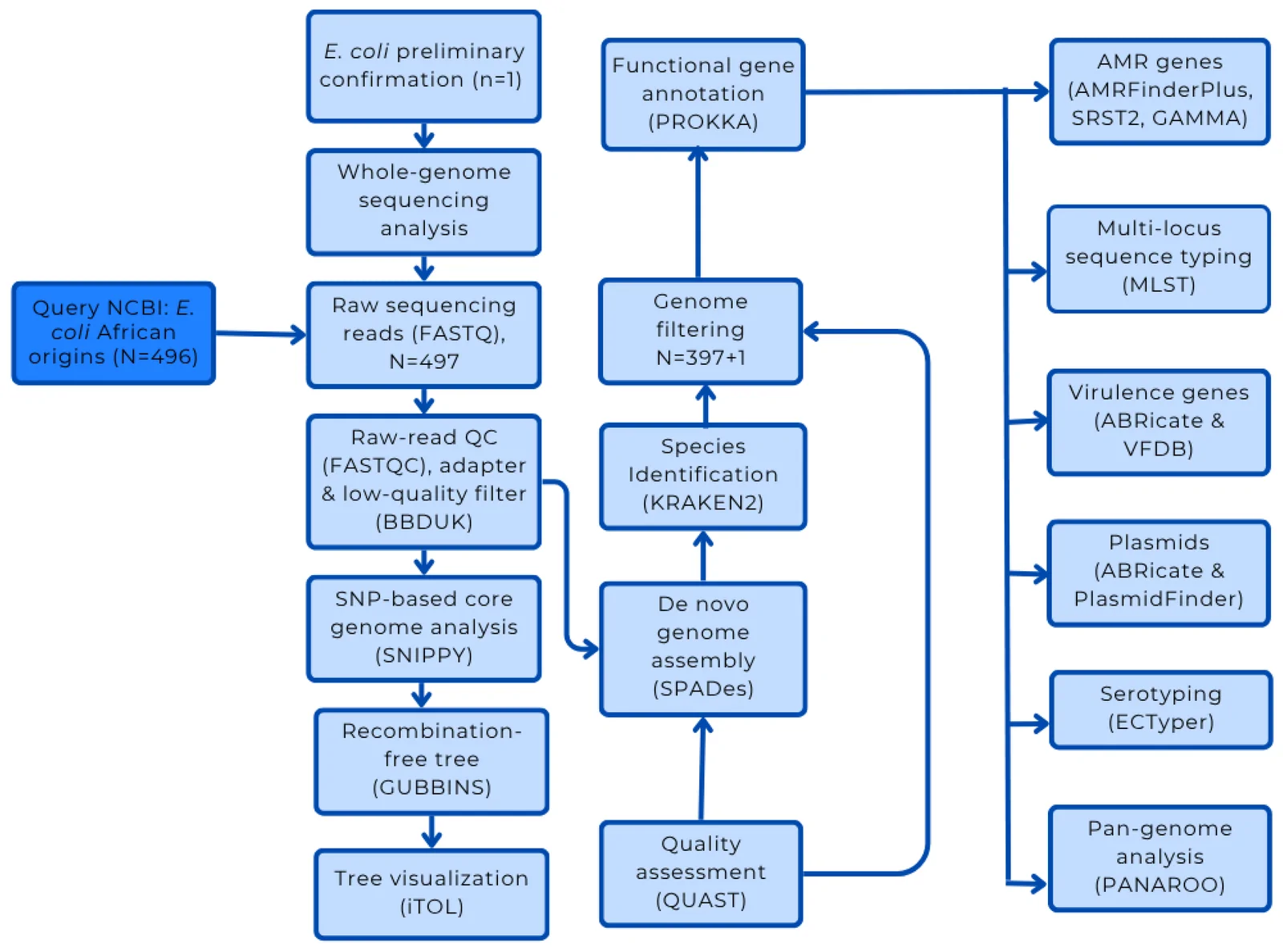
Schematic representation of the approach that was used to design this study.

Figure 1 shows the main steps of the methodology used in this study, with further details provided in the subsequent subsections.

### 2.2. Revival of Bacterial Cultures and Confirmation of Identity

The beef-derived *E. coli* isolate (EM-5_S15) was recovered from raw beef tripe purchased at a retail outlet in the King Cetshwayo District Municipality, KwaZulu-Natal Province, South Africa; sanitary practices at the point of sale were assessed as adequate at the time of sampling. The isolate was cultured on MacConkey agar (Thermo Fisher Scientific Inc., Waltham, MA, USA). Pink, lactose-fermenting presumptive colonies were selected and confirmed as *E. coli* by Matrix-Assisted Laser Desorption Ionisation Time-of-Flight (MALDI-TOF) Mass Spectrometry using the MALDI Biotyper^®^ system (Bruker Daltonics, Bremen, Germany) according to the manufacturer’s instructions [35].

### 2.3. DNA Extraction and Library Preparation

Genomic DNA was extracted from *E. coli* using the Zymo Research Bacterial and Fungal DNA Extraction Kit (Zymo Research, Irvine, CA, USA) according to the manufacturer’s instructions [36]. Sequencing libraries were prepared using an enzymatic fragmentation approach (NEBNext Ultra II FS DNA Library Prep Kit, New England Biolabs, Ipswich, MA, USA) [37]; resulting fragments were size-selected (>200 bp) using AMPure XP beads (Beckman Coulter, Brea, CA, USA) [38], end-repaired, and ligated to Illumina-specific adapter sequences. Each sample was individually indexed, followed by a second bead-based size-selection step, after which libraries were converted into Onso-compatible libraries, quantified using a fluorometric method, and diluted to a standard concentration of 4 nM prior to sequencing. Sequencing was performed at Inqaba Biotech (Pretoria, South Africa) on the PacBio Onso short-read sequencing platform (Pacific Biosciences, Menlo Park, CA, USA) using sequencing-by-binding (SBB) chemistry (300-cycle kit) according to the manufacturer’s protocol [39], generating approximately 500 Mb of 2 × 150 bp paired-end-read data per sample.

### 2.4. Genome Assembly and Annotation

A search of the NCBI SRA database on 25 September 2025 identified 496 publicly available African *E. coli* genomic datasets, which together with the beef-derived isolate yielded a working dataset of 497 genome sequences. Raw reads for the 496 SRA-derived datasets were downloaded using SRA Toolkit v3.2.0 [40] and processed alongside the beef-derived isolate using the CDCgov/phoenix v2.3.1 Nextflow pipeline [41] (Nextflow v25.10.0). Adapter and PhiX contaminant removal were performed with BBDuk from BBMap suite v39.01 [42], and low-quality reads were filtered using fastp v0.23.4 [43]. Potential contamination in filtered reads was assessed with Kraken2 v2.1.7.1 [44]. Post-trimming quality metrics, including total read counts and percentages of bases meeting Phred quality scores of Q20 and Q30, were recorded. Cleaned reads were assembled into contigs using SPAdes v4.2.0 [45,46], and contigs shorter than 500 bp were removed using the reformat.sh script from BBMap v39.01 [42]. Assembly quality was evaluated with QUAST v5.3.0 [47], and functional gene annotation was performed using Prokka v1.15.6 [48].

### 2.5. AMR Gene Prediction

Antimicrobial resistance genes were identified using the CDCgov/phoenix pipeline [41], which integrates three tools: NCBI AMRFinderPlus v4.2.25 [49], SRST2 v0.2.0 [50], and GAMMA v2.2 [51]. These tools query three curated databases: NCBI AMRFinderPlus database (version 20251203), ResFinder [52], and ARG-ANNOT [53]. Hits were retained at a minimum nucleotide identity of 90% and a minimum coverage of 80% [52].

Genes were further classified as intrinsic or acquired based on the established literature describing their genomic context: chromosomally conserved genes present regardless of resistance phenotype were designated as intrinsic, whereas genes typically disseminated via mobile genetic elements were designated as acquired (citations provided per gene in Table 1). Any statements elsewhere in this manuscript that describe a specific gene as chromosomal or plasmid-borne reflect this literature-derived genomic context rather than assembly-based confirmation in the sequenced genomes; hybrid long-read/short-read assembly and dedicated plasmid reconstruction tools (e.g., MOB-suite [54]) were not employed in this study, and such localisation attributions should therefore be read as literature-reported rather than confirmed.

### 2.6. Virulence and Pathogenicity Profiling

Virulence genes in the beef-derived isolate were identified using ABRicate v1.2.0 [64] with the *E. coli* virulence factor database (ecoli_vf) [65,66], retaining hits at a minimum identity of 90% and a minimum coverage of 80%. The virulence profiling of the beef-derived isolate was additionally performed using the VirulenceFinder web service (Center for Genomic Epidemiology, CGE) [67], where the *E. coli* species database were selected and the same identity and coverage thresholds as in ABRicate were applied. Hypervirulence-associated siderophore genes (*iucA*, aerobactin biosynthesis; *iroB*, salmochelin biosynthesis) were additionally screened across all genomes as part of the CDCgov/phoenix pipeline’s hypervirulence module. To further contextualise the beef-derived isolate’s virulence gene repertoire, the type 1 fimbrial operon (*fimA–I*), the invasion genes *ibeB*/*ibeC*, and the haemolysin *hlyE* were additionally screened across the 398 comparative genomes using ABRicate v1.2.0 with the ecoli_vf database, applying the same identity and coverage thresholds described above. Classification of genomes as enteroinvasive *E. coli* (EIEC) was performed within the CDCgov/phoenix pipeline [41].

### 2.7. Multi-Locus Sequence Typing (MLST) and Serotyping

Sequence types were assigned to all genomes using MLST v2.25.0 [68] with two schemes, the Achtman 7-locus scheme (*adk, fumC, gyrB, icd, mdh, purA, and recA*) and the Pasteur 8-locus scheme (*dinB, icdA, pabB, polB, putP, trpA, trpB, and uidA*), both based on the pubMLST database [69]. Genomes whose allelic profile did not match any existing profile were designated as novel alleles and excluded from clonal complex assignment. Clonal complex (CC) membership was determined using the goeBURST single-linkage algorithm implemented in EnteroBase [70], which groups STs that share alleles at six or more of the seven loci (single-locus variants, SLVs). Each CC is named after its predicted founder ST, the ST occupying the most ancestral node in the minimum spanning tree. For visualisation and downstream analysis, CCs represented by two or fewer genomes were collapsed into a single category designated as “Minor CC”, while genomes whose ST returned no CC assignment in EnteroBase (i.e., true singletons) were classified as “unassigned/singleton”. The in silico serotyping of the beef-derived isolate was performed using SerotypeFinder v2.0 (CGE) [71], with a minimum identity of 95% and a minimum coverage of 80%. The *fumC* and *fimH* alleles were additionally characterised using CHTyper v1.0 (CGE) [72] at the same thresholds to assign the CH type of the beef-derived isolate. The in silico serotyping of all 398 comparative genomes was additionally performed using ECTyper v1.0 [73] within the CDCgov/phoenix pipeline, with default identity and coverage thresholds; genomes for which the O-antigen or H-antigen could not be confidently resolved were flagged according to ECTyper’s built-in quality-control categories (full data in Table S7).

### 2.8. Detection of Plasmids

Plasmid replicons were screened using ABRicate v1.2.0 [64] and definitively assigned using GAMMA v2.2 [51] with the PlasmidFinder database [74] (minimum identity of 90% and length coverage of 80%), filtering out truncated or non-overlapping artefactual hits.

### 2.9. Pan-Genome Analysis

Pan-genome construction was performed using Panaroo v1.5.2 [75] with the parameters –clean-mode strict -a core –quiet -t 8. Pan-genome openness was assessed by fitting Heaps’ Law [76] to the gene cluster accumulation curve using a custom Python v3.9.7 script, modelled as:

(1) $$n=\kappa N^{\alpha },$$

where *n* is the number of gene clusters, *N* is the number of genomes sampled, κ is the scaling constant, and α is the openness parameter; α<1 indicates an open pan-genome, and α>1 indicates a closed pan-genome. The pan-genome presence/absence matrix was visualised in Python v3.9.7 using matplotlib v3.9.4 and seaborn v0.13.2 [77].

### 2.10. Core-Genome Phylogenetic Analysis

Whole-genome single-nucleotide polymorphism (SNP) analysis was performed using a Nextflow DSL2 pipeline (v25.10.0) executed on a SLURM high-performance computing (HPC) cluster. Paired-end short reads were mapped against the *E. coli* K-12 MG1655 complete genome (GenBank accession U00096.3) as the reference. Per-sample variant calling was performed using Snippy v4.6.0 [78] with default BWA-MEM alignment and quality thresholds. A core-genome alignment was constructed using snippy-core, yielding a whole-genome pseudo-alignment (core.full.aln) and an SNP-only alignment (core.aln). Prior to recombination analysis, all IUPAC ambiguity codes, gap characters, and dot characters in the core alignment were converted into N, and all bases were uppercased using awk. Groups containing fewer than three sequences were excluded, as this is the minimum required by Gubbins.

Recombination detection and masking were performed with Gubbins v3.4.1 [79] using IQ-TREE as the tree builder (-tree-builder iqtree) under the GTR + GAMMA substitution model (–model GTRGAMMA). The resulting maximum-likelihood phylogenetic tree (core.final_tree.tre) was visualised using iTOL [80]. Clonal complex annotations, country of isolation and sample type were overlaid onto the core-genome phylogeny as colour strips. Pairwise SNP distances were calculated from the recombination-filtered alignment using snp-dists v0.8.2 [81], producing an all-vs-all distance matrix. Genomic relatedness was interpreted using established thresholds: genomes differing by ≤10 SNPs were considered very closely related (potential outbreak strains), 11–25 SNPs as closely related (possible transmission linkage), and >100 SNPs as genetically distinct [82].

### 2.11. Use of Artificial Intelligence Tools

The generative AI tool Claude (Anthropic, PBC; Sonnet 4.6) was used during preparation of this manuscript to assist with language and grammar editing and to improve the clarity of the text in most sections, as well as to assist the authors in interpreting some of the results. The tool was not used for study design, data collection, generation of data or figures, or the underlying bioinformatic analyses, all of which were performed by the authors using the methods described above. All AI-assisted output was reviewed and edited by the authors, who take full responsibility for the content of this publication.

## 3. Results

### 3.1. Data Quality

Of the 496 *E. coli* sequences of African origin downloaded from the NCBI SRA, 397 were retained after genome quality filtering. Table 2 summarises the quality metrics of the beef-derived *E. coli* isolate together with the 397 NCBI SRA genome sequences. The full genomic, taxonomic, AMR gene hit, and plasmid replicon record for the beef-derived isolate is provided in Table S2.

### 3.2. AMR Determinants

The genomic screening of the beef-derived *E. coli* isolate identified both intrinsic and acquired antimicrobial resistance (AMR) determinants, summarised in Table 1 (full annotation detail in Table S1). β-Lactam-associated genes reported in the literature as chromosomal (*ampH*, *ampC1*, native penicillin-binding proteins, and the β-lactamase gene *bla*_EC-15_) were detected. *bla*_EC-15_ confers resistance to aminopenicillins such as ampicillin and amoxicillin but is not associated with extended-spectrum β-lactamase (ESBL) activity [83,84]. Intrinsic multidrug efflux pump genes *acrF* and *emrD* were also present.

Among acquired determinants, the aminoglycoside nucleotidyltransferase gene *aadA1*, encoding an ANT($3^{\prime \prime }$)-Ia enzyme, was detected and confers resistance to streptomycin and spectinomycin. The tetracycline efflux gene *tet*(B) and the streptothricin acetyltransferase gene *sat2* were also identified. No ESBL or carbapenemase genes were detected.

The acquired resistome of the beef-derived isolate thus encompasses aminoglycosides, tetracyclines, and streptothricin, while aminopenicillin resistance is conferred by the intrinsic *bla*_EC-15_ gene (reported as chromosomally encoded in the literature; see Section 2.5).

### 3.3. Virulence and Pathogenicity

Whole-genome sequencing revealed an extensive virulence gene repertoire, organised by functional category in Table 3 (reproduced with key functions in Table S4; full gene-level inventory in Table S3). Genes encoding curli fibres (*csgA–G*), type 1 fimbriae (*fimA–I*, including the mannose-binding tip adhesin *fimH*), and the *E. coli* common pilus (*ecpRABCDE*) were all detected. An intact flagellar and chemotaxis system was confirmed by the presence of *flg/flh/fli* structural genes and *cheA/cheW/cheY/cheZ/tar* signalling genes, consistent with FlhDC/FliA-regulated motility. The complete enterobactin siderophore pathway (*entA–F/S*, *fepA–G*, *fes*) was identified. Beyond colonisation-associated genes, the isolate harboured virulence determinants indicative of invasive potential. The *hlyE* gene, encoding the pore-forming haemolysin ClyA (also known as SheA), was detected at 97.93% nucleotide identity [85]. The invasion genes *ibeB* and *ibeC*, associated with penetration of brain microvascular endothelial cells in neonatal meningitis models, were also present. Non-LEE effector homologs (*espX/espL*) were detected; however, core LEE island components (*eae*, *escN*, *espA*, *espB*, *and espD*) and Shiga toxin genes (*stx*) were absent.

Pathogenicity prediction using CGE PathogenFinder classified the isolate as a probable human pathogen with a probability score of 0.944, based on detection of 532 protein families associated with human pathogenic *E. coli* compared with four non-pathogenic families. Proteome coverage against the model’s learned families was 12.13%.

### 3.4. MLST and Serotyping

MLST analysis under the Achtman seven-locus scheme assigned the beef-derived *E. coli* isolate to ST7106. Under the Institut Pasteur eight-locus scheme, the isolate yielded a novel allelic profile [*dinB*-10, *icdA*-3, *pabB*-7, *polB*-3, *putP*-41, *trpA*-1, *trpB*-4, and *uidA*-40], not previously deposited in the PubMLST database [69], with ST175 and ST1995 representing the closest matches at 75% allele identity ($\frac{6}{8}$ loci).

In silico serotyping using SerotypeFinder assigned the isolate to serotype O18ab:H11 based on an exact match to reference alleles in the database. CH typing identified *fumC* allele 11 and *fimH* allele 23, yielding CH type 11–23.

### 3.5. Plasmid Replicons

The replicon screening of the assembled genome using ABRicate against the PlasmidFinder database identified a Col(pHAD28)_1 replicon on a 2078 bp contig, with 95.7% nucleotide identity and 88.6% coverage (reference accession KU674895). Cross-tool analysis using GAMMA returned Col440I_1 as the closest replicon match, detected across two contigs: contig 223 at 90% nucleotide identity with 100% length coverage and contig 260 at 91% nucleotide identity with 96% length coverage. Both hits satisfied the applied detection thresholds (≥90% identity and ≥80% coverage). No circular plasmid element was reconstructed from the assembly.

### 3.6. Comparative Genomics

The genome data originated from 12 African countries, with Niger, presented as NE, contributing the largest proportion (177/398; 44.5%), followed by Tanzania, presented as TZ (48; 12.1%); Ethiopia, presented as ET (47; 11.8%); Egypt, presented as EGY (34; 8.5%); Tunisia, presented as TN (33; 8.3%); Mali, presented as ML (24; 6.0%); Zambia, presented as ZM (14; 3.5%); Ghana, presented as GH (8; 2.0%); Kenya, presented as KE (5; 1.3%); Nigeria, presented as NG (3; 0.8%); South Africa, presented as ZA (3; 0.8%); and Algeria, presented as DZ (2; 0.5%) (Table S8). By sample type, the collection comprised predominantly human carriage genomes (*n* = 180; 45.2%) and clinical genomes (*n* = 138; 34.7%), with additional genomes from meat (*n* = 35; 8.8%), human faecal samples (*n* = 31; 7.8%), poultry (*n* = 5; 1.3%), animal waste (*n* = 4; 1.0%), and research or laboratory sources (*n* = 3; 0.8%) and two South African genomes with unrecorded sample-type metadata (*n* = 2; 0.5%) (Table S8). The full genomic, MLST, AMR, hypervirulence, and plasmid replicon dataset for the 398 comparative African *E. coli* genomes is provided in Table S7.

#### 3.6.1. Comparative Resistome Analysis

The pan-resistome of the 398 *E. coli* genomes comprised 97 unique AMR genes spanning 14 drug classes (Table 4; full prevalence data in Table S5). β-Lactam resistance genes were the most diverse class, represented by 38 distinct determinants, followed by aminoglycosides (12 genes) and trimethoprim (10 genes). Efflux-associated genes were the most prevalent across the dataset: *emrD* was detected in 397/398 (99.7%) genomes, *acrF* in 390/398 (98.0%), and *mdtM* in 342/398 (85.9%). Among intrinsic β-lactam determinants, penicillin-binding protein genes and *ampH* were each present in 398/398 (100.0%) genomes, *ampC1* was detected in 326/398 (81.9%), and the intrinsic β-lactamase gene *bla*_EC_ (reported as chromosomally encoded in the literature; present as the allele *bla*_EC-15_ in the beef-derived isolate, at 229/398; 57.5%) was detected, across its eight alleles, in 395/398 (99.2%) genomes.

Acquired β-lactam resistance was dominated by *bla*_TEM-1_ (238/398; 59.8%) and *bla*_CTX-M-15_ (235/398; 59.0%). ESBL genes of the CTX-M family were detected in multiple variants: *bla*_CTX-M-15_ was the most prevalent (235/398; 59.0%), followed by *bla*_CTX-M-55_ (27/398; 6.8%) and *bla*_CTX-M-27_ (7/398; 1.8%). Carbapenemase genes were also detected, including *bla*_NDM-5_ (197/398; 49.5%), *bla*_OXA-1_ (119/398; 29.9%), and *bla*_OXA-48_ variants. In contrast, the beef-derived isolate harboured no acquired β-lactamase genes and lacked ESBL and carbapenemase genes; its sole β-lactam resistance determinant, *bla*_EC-15_, is intrinsic and reported in the literature as chromosomally encoded (see Section 2.5).

Among non-β-lactam acquired determinants, the sulfonamide resistance genes *sul1* (188/398; 47.2%) and *sul2* (241/398; 60.6%) were both common, with 83/398 (20.9%) genomes carrying both genes; at least one sulfonamide resistance determinant was present in 346/398 (86.9%) genomes overall. The tetracycline resistance genes *tet*(B) (191/398; 48.0%), *tet*(A) (156/398; 39.2%), *tetR* (150/398; 37.7%), and *tet*(D) (1/398; 0.3%) were widespread. Aminoglycoside-modifying enzyme genes were diverse: *aph*(6)-Id (234/398; 58.8%), *aph*($3^{\prime \prime }$)-Ib (229/398; 57.5%), and *aac*(3)-IId (132/398; 33.2%) were the most common. Trimethoprim resistance genes, predominantly *dfrA1* (106/398; 26.6%), *dfrA12* (95/398; 23.9%), and *dfrA17* (94/398; 23.6%), were detected in various combinations. The mobile colistin resistance gene *mcr-1.1* was identified in 3/398 (0.8%) genomes. Several genes were detected in a single genome only, including *bla*_NDM-19_, *bla*_NDM-20_, *bla*_OXA-244_, *bla*_CTX-M-101_, *arr-2*, *qepA4*, and *aac*(3)-IIe.

#### 3.6.2. Comparative Sequence Type and Clonal Complex Analysis

**Figure 2:**
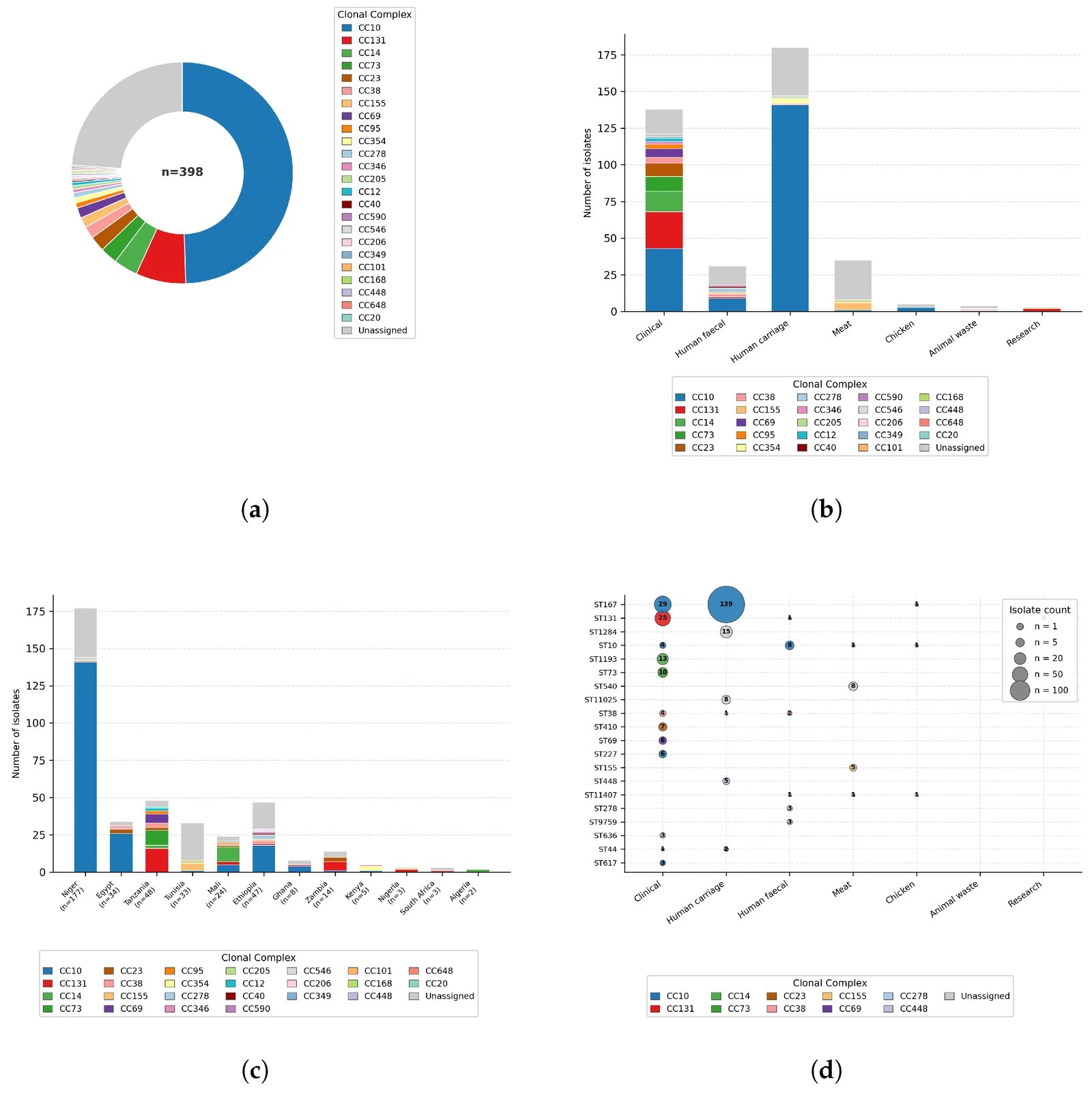
Clonal complex (CC) distribution of *Escherichia coli* genomes across source types and countries: (*n* = 398 genomes total). (**a**) Overall CC distribution; wedge area is proportional to the number of genomes per CC, and *n* = 398 denotes the total number of genomes analysed (excluding the non-African reference genome). Individual wedges for CCs each contributing ≤2% of genomes are visually condensed at this scale; their exact counts are reported in the main text (Section 3.6.2) and Table S7. (**b**) CC composition by source type; bar height = number of genomes per source category. (**c**) CC composition by country; the *n* value in parentheses beneath each country label is the total number of genomes from that country. (**d**) The 20 most frequent sequence types (STs) by source type. Bubble area and the integer inside each bubble are the number of genomes for that ST–source combination; absence of a bubble indicates zero genomes. Bubble colour denotes CC using the same key as (**a**–**c**) (legend in (**d**) shows only CCs present among these 20 STs). Grey denotes unassigned or singleton STs.

A total of 76 distinct sequence types (STs) were identified across the 398 genomes. Assignment to clonal complexes (CCs) revealed 24 named CCs, with the remaining 95 genomes (23.9%) comprising singleton or unassigned STs (Figure 2a). CC10 was the predominant complex, accounting for 197 genomes (49.5%), followed by CC131 (29/398; 7.3%), unassigned singletons (95/398; 23.9%), CC14 (14/398; 3.5%), CC73 (10/398; 2.5%), and CC23 (9/398; 2.3%). All remaining CCs each contributed fewer than 2% of the dataset.

The dominance of CC10 in the dataset is substantially driven by the composition of the Niger (NE) subset, which comprised 177 genomes (44.5% of all sequenced genomes) derived exclusively from human intestinal carriage screening (Figure 2b,c). Of these, 141 (79.7%) belonged to CC10, and 139 (78.5%) were identified as ST167, a lineage increasingly associated with carbapenem resistance in Africa [86,87]. An additional 15 Niger genomes were ST1284, also a CC10 member recovered entirely from human carriage. Consequently, the overall CC10 signal in this dataset should be interpreted with reference to this sampling structure: the high frequency of CC10 reflects in part the deliberate enrichment of ST167-positive carriage genomes in the Niger cohort rather than a uniform epidemiological representation across all countries or source types.

When the dataset is stratified by sample source type, a clear divergence in CC composition emerges between clinical and food-derived genomes (Figure 2b). Clinical specimens (*n* = 138) were dominated by established extra-intestinal pathogenic lineages, with the top five STs being ST167 (29/138; 21.0%), ST131 (25/138; 18.1%), ST1193 (13/138; 9.4%), ST73 (10/138; 7.2%), and ST410 (7/138; 5.1%). By contrast, meat-derived genomes (*n* = 35) were characterised by a strikingly different ST repertoire, where 77.1% (27/35) belonged to unassigned or singleton STs, with the most prevalent lineages being ST540 (8/35; 22.9%), ST155 (5/35; 14.3%), and ST2973 (3/35; 8.6%). Critically, none of the three dominant clinical high-risk clones, ST131, ST167, and ST1193, were detected among meat genomes, indicating a largely clonally distinct *E. coli* population at the food–human interface in this dataset.

The distribution of ST167 across source types warrants specific comment (Figure 2d). Although ST167 was the single most prevalent ST in the dataset (169/398; 42.5%), the overwhelming majority of these genomes originated from asymptomatic human carriage (139/169; 82.2%), predominantly from Niger, with only 29 ST167 genomes (17.2%) recovered from clinical (infection-associated) specimens across Egypt, Mali, Kenya, and Ethiopia. This disparity suggests that the high genomic prevalence of ST167 in African sequence repositories may partly reflect surveillance programmes targeting intestinal colonisation rather than invasive disease and underscores the importance of contextualising sequence-type frequencies against the clinical intent of the source studies.

#### 3.6.3. Comparative Serotype Analysis

In silico serotyping using ECTyper was performed across all 398 comparative genomes (full data in Table S7). A complete, high-confidence O:H serotype was resolved for 156/398 (39.2%) genomes; the O-antigen could not be confidently typed in 230/398 (57.8%) genomes (H-antigen resolved only), 9/398 (2.3%) returned an ambiguous mixed O-type call, and 3/398 (0.8%) had a non-reportable H-antigen. This high proportion of unresolved O-antigen calls is consistent with the known difficulty of assembling the *rfb* O-antigen gene cluster from short-read draft genomes, a region characterised by high GC-content variability and repetitive structure. Among the 156 genomes with a fully resolved serotype, 52 distinct O:H combinations were identified. The pandemic ExPEC clone-associated serotype O25:H4 was the most prevalent (20/156; 12.8% of fully typed genomes; 5.0% of the total dataset), consistent with the prevalence of ST131/CC131 in this dataset, followed by O75:H5 (14/156; 9.0%), O8:H9 (12/156; 7.7%), O9:H30 (9/156; 5.8%), and O6:H1 (7/156; 4.5%). The beef-derived isolate’s serotype, O18ab:H11, was not detected in any of the 397 comparative genomes, reinforcing its status as a serologically distinct lineage within this African comparative dataset.

#### 3.6.4. Comparative Virulome Analysis

The virulence gene repertoire of the beef-derived isolate was contextualised against the 398-genome comparative dataset, stratified by source metadata into clinical and non-clinical (carriage, food, and environmental) genomes, as no whole-genome sequence data specific to clinical extra-intestinal pathogenic *E. coli* (ExPEC) reference collections were available for independent comparison. The serotyping of the comparative dataset (Section 2.7) identified no genome sharing the beef-derived isolate’s exact O18ab:H11 serotype; the closest matches were a single O18:H7 genome and three O18:H4 genomes, precluding a strictly serotype-matched comparison and limiting this analysis to the broader clinical-versus-carriage stratification described below.

Hypervirulence-associated siderophore genes were screened across the 398 comparative African *E. coli* genomes as part of the CDCgov/phoenix pipeline (full data in Table S7). The aerobactin biosynthesis gene *iucA* was detected in 157/398 (39.4%) genomes, while the salmochelin biosynthesis gene *iroB* was detected in 32/398 (8.0%) genomes; both genes co-occurred in 28/398 (7.0%) genomes, and at least one marker was present in 161/398 (40.5%) genomes overall. Both genes were markedly enriched among clinical genomes relative to asymptomatic carriage genomes: *iucA* was detected in 100/138 (72.5%) clinical genomes compared with 17/180 (9.4%) human carriage genomes, and *iroB* showed a similar pattern (23/138; 16.7% clinical versus 0/180 carriage). *iroB* was also comparatively common among meat-derived genomes (8/35; 22.9%) and among genomes from Tanzania (18/48; 37.5%). This clinical enrichment is consistent with the established role of aerobactin and salmochelin as high-affinity iron-acquisition systems that enhance extra-intestinal survival and invasiveness in ExPEC lineages. Neither *iucA* nor *iroB* was detected in the beef-derived isolate (EM-5_S15), consistent with its narrower virulence profile centred on the enterobactin siderophore system, type 1 fimbriae, curli fibres, and the invasion genes *ibeB*/*ibeC* (Section 3.3), rather than the aerobactin/salmochelin systems more typical of the high-risk clinical lineages in the comparative dataset.

**Figure 3:**
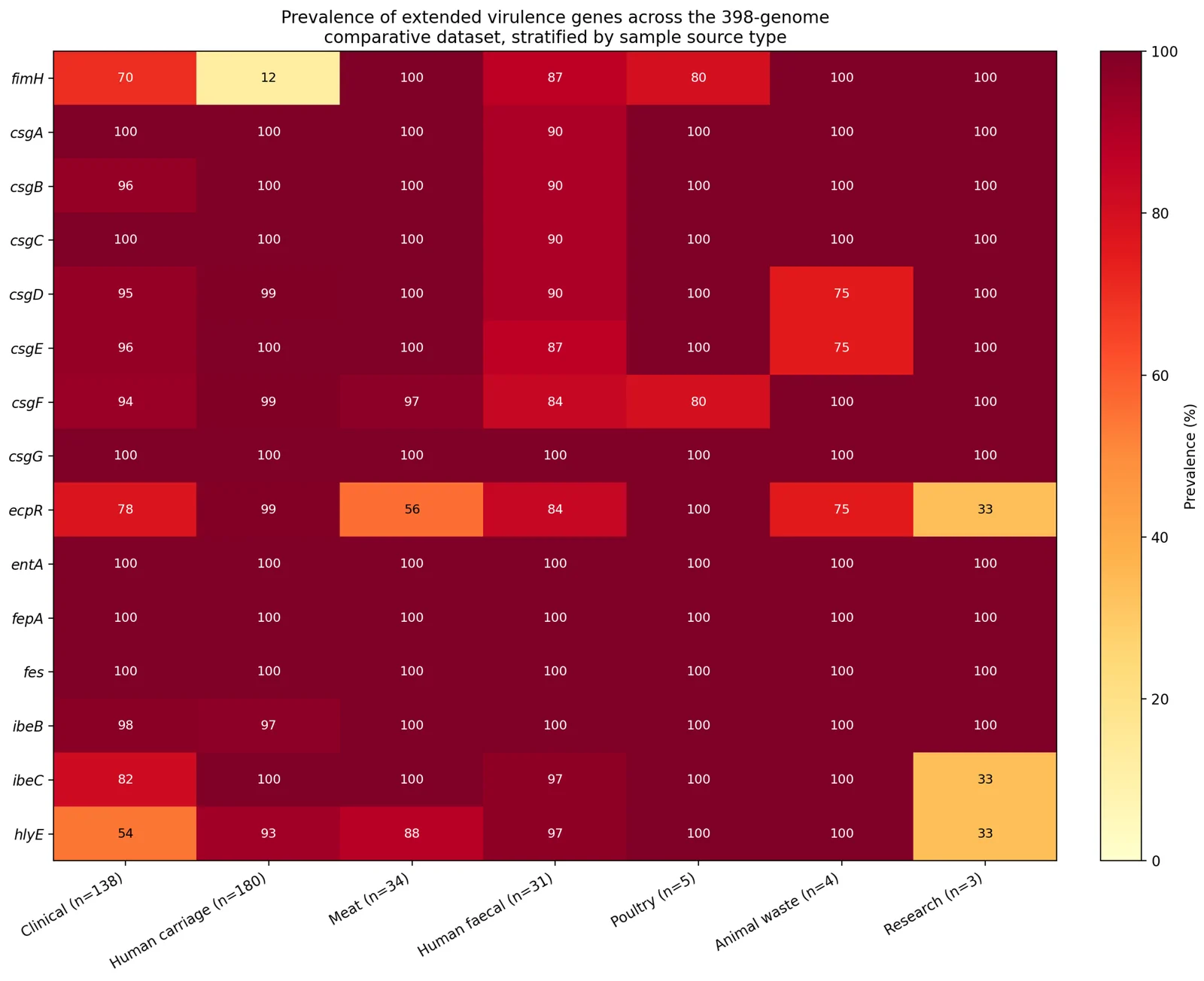
Prevalence (%) of extended virulence genes (*fimH*, curli operons *csgA–G*, *ecpR*, enterobactin genes *entA/fepA/fes*, invasion genes *ibeB/ibeC*, and *hlyE*) across the 398 comparative African *E. coli* genomes, stratified by sample source type. Values are the percentage of genomes within each category carrying the gene. Full per-category gene counts are provided in Table S9.

Extending this comparison to the isolate’s broader ABRicate/ecoli_vf virulence repertoire (Section 3.3), prevalence of the type 1 fimbrial adhesin gene *fimH*, curli biosynthesis genes (*csgA–G*), the *E. coli* common pilus gene *ecpR*, the enterobactin siderophore genes (*entA*, *fepA*, and *fes*), and the invasion/haemolysin genes *ibeB*, *ibeC*, and *hlyE* was determined across all sample source categories in the 398-genome dataset (Figure 3; full per-category counts in Table S9). The type 1 fimbrial adhesin gene *fimH* was clinically enriched (97/138; 70.3% clinical versus 22/180; 12.2% carriage). The enterobactin siderophore genes and curli biosynthesis genes, by contrast, were near-universal across the dataset (≥97% in both groups) and therefore uninformative for clinical differentiation, while the invasion genes *ibeB*/*ibeC* and the haemolysin *hlyE* showed no clinical enrichment (*ibeB*: 97.8% clinical versus 97.2% carriage; *ibeC*: 81.9% clinical versus 100% carriage; *hlyE*: 54.3% clinical versus 93.3% carriage; i.e., more prevalent among carriage genomes), reinforcing the lack of a validated ExPEC virulence gene consensus noted among the limitations in the Discussion Section. Thus, of the isolate’s virulence gene repertoire, only the aerobactin/salmochelin siderophore genes and *fimH* show a clear clinical association in this comparative dataset, while *ibeB*, *ibeC*, and *hlyE* do not discriminate between clinical and carriage genomes and therefore do not, on their own, support an ExPEC-specific interpretation of the beef-derived isolate’s virulence gene content.

To further characterise the dominant ST167 lineage, its AMR and virulence gene content was compared against the remaining 229 comparative genomes. ST167 genomes carried a substantially higher carbapenemase burden: 156/169 (92.3%) harboured at least one carbapenemase gene, predominantly *bla*_NDM-5_ (155/169; 91.7%), compared with only 45/229 (19.7%) of the remaining genomes (*bla*_NDM-5_, 42/229; 18.3%), confirming ST167 as the principal vehicle for carbapenem resistance in this dataset. ST167 also showed markedly higher carriage of *bla*_CTX-M-15_ (136/169; 80.5% versus 99/229; 43.2%) and the trimethoprim resistance genes *dfrA1* (71/169; 42.0% versus 35/229; 15.3%) and *dfrA12* (78/169; 46.2% versus 17/229; 7.4%), whereas *sul2* (3/169; 1.8% versus 66/229; 28.8%) and *mcr-1.1* (0/169 versus 3/229; 1.3%) were comparatively rare. In contrast, the hypervirulence-associated siderophore genes *iucA* and *iroB* described above were detected far less frequently in ST167 than in the rest of the dataset (*iucA*, 14/169; 8.3% versus 143/229; 62.4%; *iroB*, 0/169 versus 32/229; 14.0%), despite a higher mean AMR/hypervirulence gene count per genome overall (21.4 versus 18.0). Together, these findings indicate that ST167 in this dataset represents a carbapenemase- and ESBL-driven multidrug-resistant lineage rather than a hypervirulence-associated one, consistent with its established role as a major global vehicle for *bla*_NDM-5_ dissemination on IncX3 plasmids.

#### 3.6.5. Pan-Plasmid Replicon Analysis

PlasmidFinder analysis of the 398 *E. coli* genomes identified 51 unique plasmid replicon types (Table 5). IncF-type replicons were the most diverse and prevalent family, comprising 18 distinct replicon types. Among these, IncFIB (AP001918) was the most frequently detected (199/398; 50.0%) genomes, followed by IncFIA (192/398; 48.2%) and IncFII (112/398; 28.1%).

Colicin-producing (Col) replicons were the second most diverse group, with 11 types. Col(BS512) was found in 182/398 (45.7%) genomes, Col156 in 157/398 (39.4%), and Col(pHAD28) in 121/398 (30.4%). Additionally, IncI1-I(Alpha) was detected in 127/398 (31.9%) genomes. While IncX-type replicons were present in 130/398 (32.6%) genomes collectively, IncX3 was the most common subtype (115/398; 28.9%). Lower prevalence included IncY (26/398; 6.5%) and various IncHI types, such as IncHI2 and IncHI2A (8/398 each; 2.0%), and IncHI1A and IncHI1B (2/398 each; 0.5%). Notably, the IncHI1B (pNDM-CIT) replicon was identified in a single genome.

### 3.7. Pan-Genome Structure

**Figure 4:**
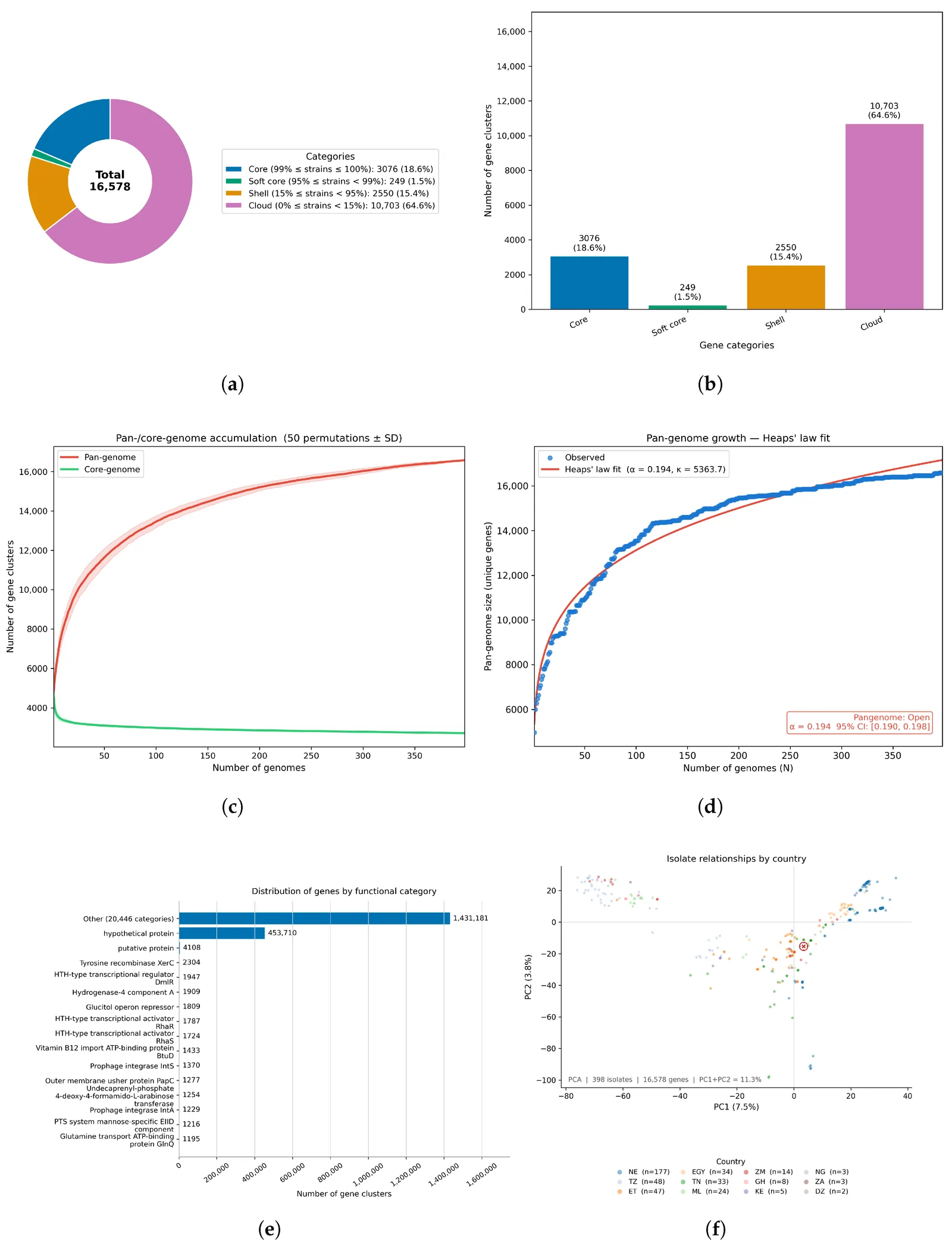
Pan-genome composition: (**a**) Core-, shell-, and cloud-genome distribution. (**b**) Summary statistics. (**c**) Pan-genome accumulation curve. (**d**) Pan-genome Heaps’ Law curve. (**e**) Pan-genome functional categories. (**f**) PCA plot of gene presence/absence in pan-genome.

Pan-genome analysis of 398 *E. coli* genomes using Panaroo v1.5.2 identified a total of 16,578 gene clusters (Figure 4a,b). The pan-genome was partitioned into four categories: 3076 (18.6%) core genes (present in ≥99% of genomes), 249 (1.5%) soft-core genes (present in 95–99% of genomes), 2550 (15.4%) shell genes (present in 15–95% of genomes), and 10,703 (64.6%) cloud genes (present in <15% of genomes).

The pan-genome accumulation curve showed a continuously rising trajectory with no sign of saturation across the 398 studied genomes (Figure 4c). In contrast, the core-genome curve declined and stabilised at approximately 2700 gene clusters. Fitting Heaps’ Law to the pan-genome growth data yielded an openness parameter of α=0.194 (95% CI: [0.190, 0.198]; κ=5363.7) (Figure 4d), confirming that the *E. coli* species possesses an open pan-genome (α<1).

Functional annotation of pan-genome gene clusters indicated that the largest categories were hypothetical proteins (419,480 instances) and genes assigned to other 20,799 functional categories (1,469,128 instances) (Figure 4e). Among the annotated groups, prophage-associated genes were highly abundant, with prophage integrases IntS (1370 instances) and IntA (1229 instances) being among the most frequently represented clusters. Other abundant functional groups included tyrosine recombinase XerC (2167 instances), the multidrug efflux RND transporter permease subunit AcrB (1194 instances), and the HTH-type transcriptional activators RhaR and RhaS (1459 and 1324 instances, respectively).

Principal component analysis (PCA) of the gene presence/absence matrix revealed substantial genomic diversity across the 398 genomes, with PC1 and PC2 explaining 7.5% and 3.8% of the total variance, respectively (Figure 4f). The genomes were widely distributed broadly across the principal component space, with no clear clustering pattern observed.

Pan-genome analysis identified nine gene clusters unique to the beef-derived *E. coli* isolate (Table 6). Eight of these clusters were annotated as hypothetical proteins, while the remaining cluster was assigned as a Type II DNA methyltransferase (M1.BsuMI), a component of bacterial restriction–modification systems.

### 3.8. Core-Genome Analysis

**Figure 5:**
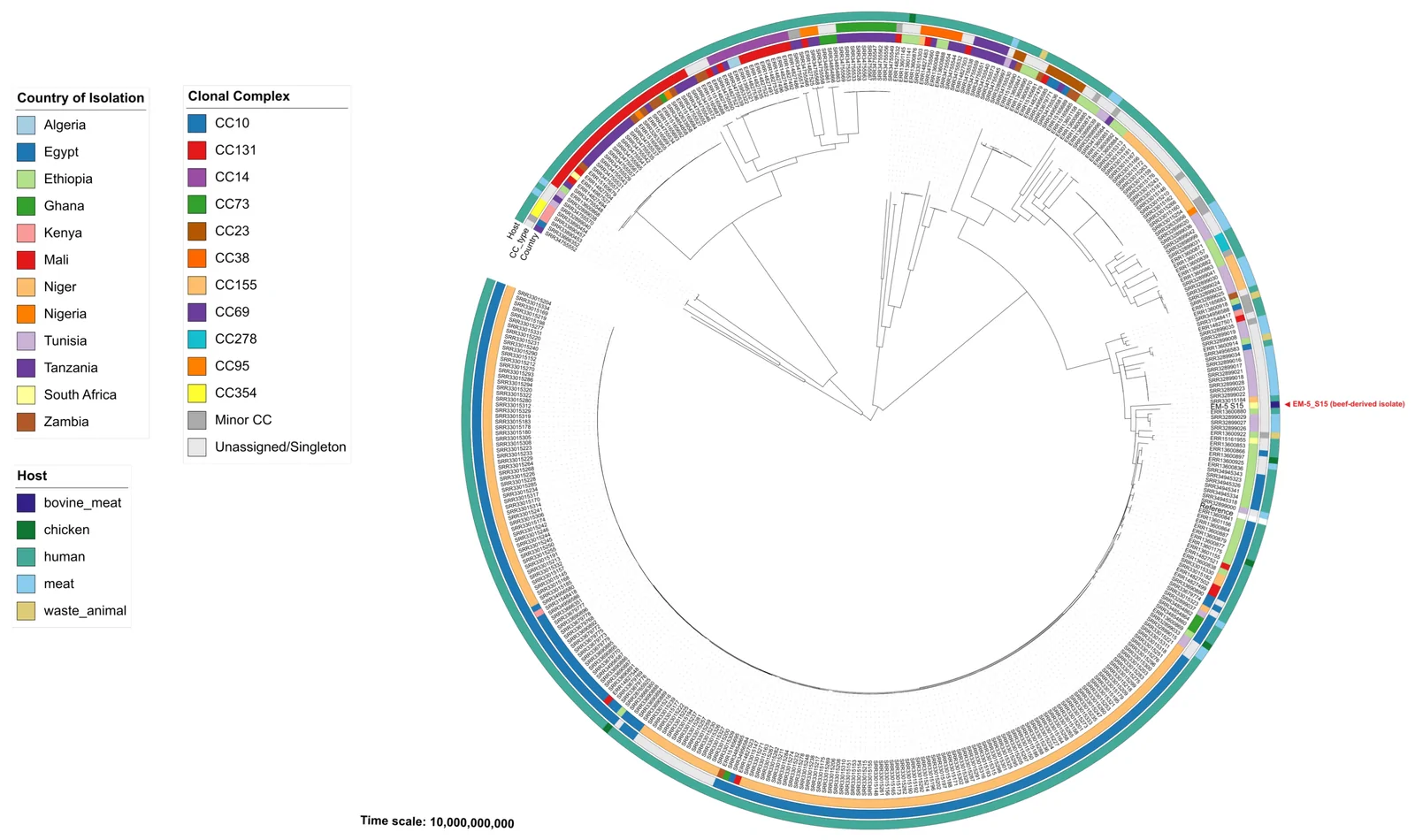
Core-genome maximum-likelihood phylogenetic tree. Colour strips (inner to outer) show country of isolation, clonal complex (CC) type and host source as defined in the legend on the left. The beef-derived isolate from this study (EM-5_S15) is marked with a red arrow and label.

Core-genome analysis of the 397 NCBI SRA *E. coli* genome data, the beef-derived *E. coli* isolate, and the K-12 MG1655 complete genome: GenBank accession U00096.3 identified 1,506,494 SNPs across the phylogeny. Gubbins detected 25,706 recombination blocks totalling 57.7 Mbp (median block length: 534 bp; range: 3–150,506 bp). The core-genome phylogenetic tree is shown in Figure 5.

The beef-derived isolate (EM-5_S15) accumulated 13,063 SNPs along its evolutionary branch and showed a recombination ratio of $\frac{\rho }{\theta }=0.0657$, slightly above the population median ($\frac{\rho }{\theta }=0.0513$). A total of 358 recombination blocks were detected in the EM-5_S15 lineage.

After removing recombinant regions, pairwise SNP distances from EM-5_S15 to all other genomes ranged from 1629 to 42,724 SNPs (median: 2412 SNPs), compared with a population-wide median pairwise distance of 9181 SNPs (Table 7).

## 4. Discussion

### 4.1. Beef-Derived E. coli Isolate

The whole-genome sequencing of the beef-derived *E. coli* isolate revealed a genomic profile characterised by a clinically relevant serotype, a novel sequence type, an extensive virulence gene repertoire, and acquired antimicrobial resistance determinants. Whole-genome sequencing-based characterisation of food-associated *E. coli* in South Africa remains limited, and African food-chain genomes are substantially underrepresented in global genomic databases. The present study addresses this gap by providing the first WGS-based genomic characterisation of a retail beef-derived *E. coli* isolate from South Africa, contextualised within a comparative dataset of 397 publicly available African genomes. Taken together, the findings demonstrate that WGS reveals a depth of pathogenic and resistance potential in food-associated *E. coli* that phenotypic methods alone cannot resolve.

The resistome of the beef-derived *E. coli* isolate was characterised by acquired resistance confined to narrow-spectrum agents, namely, aminoglycosides, tetracyclines, and streptothricin, alongside intrinsic aminopenicillin resistance conferred by the β-lactamase gene *bla*_EC-15_ (reported as chromosomally encoded in the literature). This resistance profile is consistent with the historically pervasive selective pressures exerted by these drug classes in livestock production systems, where aminopenicillins and tetracyclines remain among the most heavily consumed antimicrobials in food-producing animals [88]. Tetracycline, aminoglycoside, and beta-lactam resistance genes are, indeed, the most abundant classes routinely detected in the bovine faecal resistome, even in the absence of direct therapeutic exposure [89]. The co-occurrence of streptothricin resistance is noteworthy, as *sat* genes encoding streptothricin acetyltransferases were first mobilised following the agricultural use of nourseothricin as a growth promoter and have since become widely disseminated on integron-borne gene cassettes in Enterobacterales from food-animal and environmental reservoirs [90]. Crucially, the absence of extended-spectrum beta-lactamase (ESBL) and carbapenemase determinants distinguishes this isolate from the multidrug-resistant lineages that dominate the comparative dataset. ESBL-producing *Escherichia coli* are increasingly reported along the farm-to-fork continuum and are recognised as clinically relevant reservoirs capable of foodborne transmission to humans [91], while carbapenemase-producing Enterobacterales, although still rare in livestock, represent an escalating One Health concern when detected in food-animal settings [92]. The restricted resistance repertoire of the present isolate therefore suggests a lower potential for treatment compromise relative to the ESBL- and carbapenemase-harbouring clones that typify high-risk nosocomial and community-associated lineages.

The intrinsic efflux pump genes *acrF* and *emrD* reflect baseline resistance capacity inherent to the *E. coli* core genome rather than acquired resistance. The overall acquired resistance profile of the isolate is comparatively limited, encompassing aminoglycosides, tetracyclines, and streptothricin, in addition to intrinsic aminopenicillin resistance conferred by *bla*_EC-15_. These classes are consistent with those commonly reported in beef-associated *E. coli* [17,18]. However, the co-occurrence of resistance and virulence determinants in a food-derived isolate remains of significant public health concern, as *E. coli* is a well-recognised vehicle for the horizontal transfer of resistance genes across animal and human reservoirs [28].

The virulence gene repertoire identified in this isolate includes genes with established roles in extra-intestinal colonisation and, in some lineages, invasive disease; however, as detailed below, the present data do not support assigning this isolate a definitive extra-intestinal pathogenic *E. coli* (ExPEC) pathotype. Colonisation-associated genes, including type 1 fimbriae (*fimA–I*, *fimH*), curli fibres (*csgA–G*), and the *E. coli* common pilus (*ecpRABCDE*), collectively facilitate adhesion to host epithelial surfaces and promote biofilm formation, functions relevant to both intestinal and extra-intestinal colonisation [93,94]. The complete enterobactin siderophore system (*entA–F/S*, *fepA–G*, *fes*) further equips the isolate for survival under iron-restricted host conditions; this mechanism is broadly conserved across *E. coli* pathotypes and commensal lineages alike (Section 3.6.4) rather than being ExPEC-specific in isolation [95,96].

The isolate additionally carries *ibeB* and *ibeC*, invasion genes associated in some lineages with penetration of brain microvascular endothelial cells and classically linked with neonatal meningitis-associated *E. coli* (NMEC) [97,98], and *hlyE* (*clyA/sheA*), detected at 97.93% nucleotide identity, encoding a pore-forming cytolysin implicated as a virulence factor in a subset of pathogenic *E. coli* lineages [85,99,100]. However, the comparative analysis of these same genes across the 398-genome African dataset (Section 3.6.4) found no enrichment of *ibeB*, *ibeC*, nor *hlyE* in clinical genomes relative to carriage genomes; *hlyE* in particular was more prevalent among carriage genomes (93.3%) than clinical genomes (54.3%). Combined with the absence of any close core-genome relationship with a clinical isolate (minimum pairwise distance of 1629 SNPs to the nearest comparative genome) and the absence of in vivo pathogenicity testing, these genes alone are insufficient to assign this isolate an ExPEC or NMEC pathotype, consistent with the lack of scientific consensus on a minimum virulence gene set defining ExPEC [95]. Although non-LEE effector homologs (*espX/espL*) were present, the absence of the core LEE island genes (*eae*, *escN*, *espA*, *espB*, and *espD*) and Shiga toxin genes (*stx*) argues against EPEC or EHEC classification, as these pathotypes are defined by the presence of the LEE-encoded attaching-and-effacing machinery and/or *stx* [101,102]. Taken together, the isolate’s virulence gene repertoire indicates colonisation capacity and carriage of individual genes with documented invasive roles in other lineages but does not, based on current evidence, support assignment to a defined extra-intestinal pathotype.

Under the Achtman seven-locus scheme, the beef-derived isolate was assigned to ST7106, a sequence type with no prior characterisation in the published literature, suggesting that this lineage falls outside the scope of current global genomic surveillance. This genetic distinctiveness was reinforced by the Institut Pasteur eight-locus scheme, under which no exact ST could be assigned owing to a novel allelic profile [103,104]. The detection of an uncharacterised sequence type in retail beef from South Africa adds to a growing body of evidence that food production environments harbour a broader diversity of *E. coli* sequence types than routine surveillance captures, including lineages with potential clinical relevance [105,106].

The O18ab:H11 serotype is well documented in the literature among ExPEC strains responsible for neonatal meningitis, septicaemia, and urinary tract infections [107,108], although as discussed above, the present isolate’s own comparative and phylogenetic data do not independently confirm an ExPEC pathotype for this specific genome. Of particular One Health relevance, O18 strains of avian and human origin have been shown to employ overlapping pathogenic mechanisms, including invasion of brain microvascular endothelial cells [108], suggesting that food-animal reservoirs may harbour strains with zoonotic potential. The high pathogenicity probability returned by PathogenFinder (0.944), informed by the detection of 532 pathogen-associated protein families against only four non-pathogenic families, indicates that the isolate’s protein content resembles that of known human pathogens. However, the 12.13% proteome coverage limits the sensitivity of this computational prediction, and, absent in vivo pathogenicity testing or a confirmed ExPEC pathotype (see above), this score should be interpreted as a broad genomic signal rather than confirmation of clinical pathogenicity.

Replicon screening revealed sequence signals consistent with a ColE1-family plasmid, though no intact circular backbone was recovered in the short-read assembly. The observed inter-tool discrepancy where ABRicate reported Col(pHAD28)_1 while GAMMA assigned the locus to Col440I_1 reflects overlapping sequence homology within the ColE1-like superfamily. Alignment analysis confirmed that Col(pHAD28)_1 represented a secondary, partial alignment on scaffold 260, which GAMMA correctly resolved and filtered as a divergent Col440I_1 sequence variant. Supporting this, the 9–10% nucleotide divergence relative to the Col440I_1 reference across both scaffolds (223 and 260), combined with near-complete target coverage (96–100%), indicates the presence of a single, divergent ColE1-family replicon lineage. Failure to assemble a closed plasmid is a well-documented limitation of short-read *de-novo* assembly, particularly for small, high-copy ColE1-type replicons whose dense, repetitive regulatory and mobilisation elements frequently collapse assembly graphs [109]. Consequently, the lack of a closed contig does not preclude the existence of a functional, autonomous plasmid in the native isolate. Definitive structural resolution, including verification of circularity and identification of associated cargo genes, will require long-read or hybrid sequencing in combination with graph-aware plasmid reconstruction tools such as MOB-suite [54] or Unicycler. Crucially, because an intact plasmid backbone was not closed, the possibility that these detected resistance and virulence determinants are harboured within chromosomally integrated elements (e.g., transposons or integrative mobilisable elements) cannot be ruled out. This distinction carries significant functional implications: while chromosomal integration typically enhances the evolutionary stability and vertical inheritance of accessory genes, plasmid carriage facilitates rapid, horizontal dissemination across diverse lineages [110].

### 4.2. Comparative Analysis

The pan-resistome analysis of 398 African *E. coli* genomes revealed a diverse and clinically alarming AMR gene repertoire, reflecting the escalating continental resistance burden outlined in the Introduction. The predominance of *bla*_CTX-M-15_ (59.0%) aligns with the global dissemination of this ESBL variant, which is the most frequently reported CTX-M type worldwide. This widespread distribution has been extensively documented in both clinical and community settings across Africa [111]. The co-occurrence of *bla*_TEM-1_ at comparable prevalence indicates that a substantial proportion of genomes harbour multiple β-lactamase genes, a signature of the multidrug-resistant *E. coli* lineages that increasingly dominate African clinical landscapes.

Of particular concern is the high prevalence of carbapenemase genes, most notably *bla*_NDM-5_, detected in nearly half of the genomes (49.5%). NDM-producing *E. coli* pose a critical public health threat, given that carbapenems represent last-resort agents for the treatment of severe Gram-negative infections [86,112]. The co-detection of OXA-48-like carbapenemases alongside NDM variants within this dataset raises the possibility of dual carbapenemase carriage, a genotype that would severely constrain therapeutic options [113]. Although detected at low prevalence (0.8%), the identification of *mcr-1.1* is equally significant: colistin frequently serves as the sole remaining treatment option for carbapenem-resistant Enterobacterales, and the mobilisable nature of *mcr* genes threatens to erode this final therapeutic line [114].

The aminoglycoside resistome was defined by the frequent co-occurrence of *aph*(3”)-Ib and *aph*(6)-Id, a gene pair characteristically co-located on class 1 integrons and encoding streptomycin resistance [57]. This pattern, together with the widespread presence of sulfonamide (*sul1*/*sul2*) and trimethoprim (*dfrA*) resistance determinants, points to a high burden of integron-mediated multidrug resistance across the dataset. Notably, this genetic architecture facilitates the co-selection and co-transfer of resistance to multiple drug classes during a single selective event.

In contrast, the beef-derived isolate exhibited a markedly more restricted resistome, lacking ESBL, carbapenemase, and colistin resistance determinants. While this profile represents a lower immediate clinical risk than the extensively resistant genotypes prevalent in the broader dataset, it nonetheless reflects ongoing selective pressure from antimicrobial use within the beef production chain [15,16,17,18].

The dominance of IncF-family replicons across the dataset is consistent with the well-established role of IncF plasmids as the principal vehicles for AMR and virulence gene dissemination in *E. coli*, particularly within pandemic lineages such as ST131 [115,116]. The high prevalence of IncX3 replicons (28.9%) is especially notable, as this replicon type has emerged as a key vector for *bla*_NDM-5_ dissemination across phylogenetically diverse sequence types [117,118], consistent with reports of active horizontal transfer of carbapenem resistance genes in African and Asian settings [112]. The abundance of small Col-type replicons likely reflects their ecological role as mobilisable accessory elements that frequently co-transfer with larger conjugative plasmids, thereby enhancing the persistence and stability of resistance plasmid populations within the host bacterial community [119].

High-risk *E. coli* clones accounted for over two-thirds of genomes (274/398; 68.8%), underscoring the substantial public health burden that these lineages impose across the African continent.

CC10 was the most prevalent clonal complex (197/398; 49.5%), the only CC recovered from all 12 countries and all sample source categories, consistent with its status as a globally versatile, host-generalist lineage [120,121]. Within CC10, ST167 predominated, particularly among genomes from Niger and Egypt. This lineage has emerged as a principal carrier of *bla*_NDM-5_ on IncX3 plasmids and a major driver of carbapenem-resistant *E. coli* dissemination across Africa, Asia, and Europe [86,87,117,122]. Its high prevalence here likely reflects the composition of publicly available African genomes, which are disproportionately derived from carbapenem-resistance-focused studies, but nonetheless confirms the clinical prominence of this lineage on the continent.

ST131 (CC131), detected across seven countries, is a pandemic ExPEC clone associated with fluoroquinolone resistance and *bla*_CTX-M-15_ [123], and its recovery from both clinical and community settings is consistent with its capacity for extra-intestinal infection and asymptomatic colonisation [124]. ST1193 (CC14), the third most prevalent clinical lineage, is a fluoroquinolone-resistant sub-lineage that has expanded globally from within the CC14 radiation [125,126]. Two further ExPEC complexes were also represented: CC73, which is linked with pyelonephritis and bacteraemia, and CC69, which is associated with trimethoprim–sulfamethoxazole resistance [121,124].

A clear clonal separation was evident between clinical and food-animal populations. Meat-derived genomes were dominated by ST540 (22.9%), ST155 (14.3%), and ST2973 (8.6%). These lineages are largely absent from the clinical fraction. ST155 (CC155) has been associated with mobile colistin resistance in livestock [127], and both ST540 and ST155 have been reported in African meat and livestock environments [128]; ST2973, to our knowledge, has not been previously characterised in this context.

The exception was CC38, detected in both meat and human genomes, consistent with its characterisation as an emerging ESBL-associated clade bridging clinical and food production settings [129,130]. The absence of these dominant clinical clones (ST131, ST167, and ST1193) in meat genomes indicates that the two populations remain largely clonally distinct. However, the presence of shared IncF and IncI1 replicons suggests that resistance determinants may traverse the human–food-animal interface via conjugative plasmid transfer independently of clonal spread. This points to a One Health convergence operating at the mobile genetic element level rather than the lineage level.

The position of the beef-derived ST7106 isolate outside all recognised clonal complexes in this dataset further illustrates that clinically relevant virulence potential can reside in lineages not captured by resistance-focused genomic surveillance.

The open pan-genome structure (α=0.194) and bimodal gene frequency distribution are consistent with the well-established genomic plasticity of *E. coli*, which possesses one of the largest known bacterial pan-genomes [76,131]. The continuously rising pan-genome curve, unsaturated across 398 genomes, indicates that the African *E. coli* accessory gene reservoir remains incompletely sampled, with cloud genes (64.6%) constituting the dominant compartment. In contrast, the core genome stabilised at approximately 2700 gene clusters, confirming that this dataset is sufficiently large to define the conserved functional backbone of the species. The large disparity between the core genome (3325 genes including soft core) and the total pan-genome (16,578 clusters) underscores that the vast majority of genetic diversity resides within the accessory and cloud compartments. These fluid genomic sectors act as the primary reservoir through which resistance and virulence determinants are acquired and disseminated via horizontal gene transfer [132].

Among the nine gene clusters unique to the beef-derived isolate, most encoded hypothetical proteins; the exception was a TypeII methyltransferase (M1.BsuMI; Table 6), a component of a restriction–modification system that protects endogenous DNA from cleavage and defends against bacteriophage predation [133,134]. The presence of this system may influence the capacity of this lineage to acquire novel mobile genetic elements, while the hypothetical clusters warrant further investigation as potential adaptations to the food production environment.

Extensive recombination was detected across the dataset (25,706 blocks; 57.7 Mbp), reinforcing the necessity of recombination-aware phylogenetic methods for *E. coli* [79,135]. For the beef-derived isolate EM-5_S15, the accumulation of 13,063 SNPs along its evolutionary branch reflects substantial independent divergence from its last common ancestor within the dataset. Its recombination-to-mutation ratio (ρ/θ=0.0657), marginally above the population median (0.0513), indicates that point mutation rather than recombination is the dominant evolutionary force shaping this lineage. The phylogenetic placement of EM-5_S15 therefore reflects genuine evolutionary divergence rather than an artefact of lateral gene transfer, lending confidence to its classification as a genomically distinct lineage [132,135].

The detection of a genomically distinct *E. coli* lineage carrying acquired resistance genes and individual virulence genes with documented invasive roles in other lineages, in South African retail beef, carries One Health relevance. The O18ab:H11 serotype has documented pathogenic associations in both human and avian hosts in the wider literature, although as discussed above, the present isolate’s own comparative and phylogenetic data do not support a confirmed ExPEC/NMEC pathotype assignment. These findings nonetheless illustrate that retail meat can harbour genomically novel *E. coli* lineages carrying genes of documented invasive potential in other contexts, underscoring the value of WGS-based surveillance for detecting such lineages regardless of whether a definitive clinical pathotype can be assigned from genomic data alone.

This study has several limitations. First, the beef-derived component is a single isolate, precluding inference about the prevalence of this lineage, serotype, or resistance profile within the South African beef supply. These findings should therefore be read as a case description rather than a prevalence estimate. Second, the 398-genome comparative dataset reflects the current composition of publicly available African *E. coli* sequences rather than a representative continental survey. A single Niger study targeting carbapenem-resistant carriage isolates contributes 177/398 (44.5%) of genomes, inflating the apparent prevalence of CC10, ST167, and *bla*_NDM-5_/IncX3 (Section 3.6.2) relative to the true African epidemiological picture; this underscores the need for coordinated, standardised genomic surveillance continent-wide. Third, resistance and virulence profiles were genotypically predicted rather than phenotypically confirmed: no antimicrobial susceptibility testing (e.g., broth microdilution or disk diffusion) was performed. Genotype-based AMR prediction shows high concordance with phenotypic testing [49], but *bla*_EC-15_ detection indicates genetic potential rather than a confirmed resistant phenotype. Likewise, the invasive capacity conferred by *ibeB*/*ibeC* was inferred from sequence homology rather than confirmed with in vitro assays (e.g., brain microvascular endothelial cell invasion models). Fourth, as discussed in Section 4.1, individual genes with documented ExPEC/NMEC-associated roles were detected in the isolate, but a definitive ExPEC/NMEC pathotype assignment is not supported by gene homology or PathogenFinder’s computational score alone. This reflects the lack of a consensus ExPEC virulence gene set (unlike STEC/EPEC) and the absence of in vivo pathogenicity testing. Relatedly, the comparative virulome analysis (Section 3.6.4) was limited to clinical-versus-carriage stratification, as no genome sharing the isolate’s O18ab:H11 serotype nor a dedicated clinical ExPEC reference collection was available. In vivo pathogenicity testing, serotype-matched clinical comparison, and phenotypic validation of the resistance and virulence profiles reported here represent priorities for future work. Fifth, this study was designed as a genomic surveillance and characterisation study rather than a source-attribution or outbreak investigation. Contamination-source attribution along the farm-to-fork continuum (e.g., slaughter, transport, or handling prior to retail) was not undertaken, and no clinical history applies, as the isolate was not linked with a reported human illness episode.

The African continent remains substantially underrepresented in global genomic surveillance of food-associated *E. coli*, and South Africa in particular lacks published WGS data on *E. coli* from retail beef. By combining comparative analysis of 398 African genomes with the genomic characterisation of a novel isolate from an otherwise unmonitored reservoir, this study begins to address that gap.

## 5. Conclusions

This study provides the first WGS-based genomic characterisation of a retail beef-derived *E. coli* isolate from South Africa. The isolate was assigned to the novel sequence type ST7106 and serotype O18ab:H11 and carried a narrow acquired resistome (aminoglycosides, tetracyclines, and streptothricin), the intrinsic β-lactamase gene *bla*_EC-15_ (reported as chromosomally encoded in the literature), and individual virulence genes (*ibeB*, *ibeC*, and *hlyE*) with documented invasive roles in other *E. coli* lineages. However, comparative and phylogenetic analyses did not support assigning this isolate a confirmed extra-intestinal pathogenic *E. coli* (ExPEC) pathotype, underscoring that gene presence alone, without clinical enrichment or in vivo confirmation, is insufficient for pathotype assignment. This study also makes a broader contribution through the comparative genomic analysis of 398 African *E. coli* genomes spanning 12 countries. This analysis revealed a substantial continental pan-resistome of 97 unique resistance genes across 14 drug classes, concentrated among recognised high-risk clinical clones (ST167, ST131, ST1193, and ST410); an open pan-genome structure (Heaps’ law coefficient α=0.194; 16,578 gene clusters); and shared plasmid replicons (IncF and IncI1) spanning clinically and food-animal-associated genomes independent of clonal lineage. Clinical and food-animal *E. coli* populations formed clearly separated clonal groups, within which the beef-derived isolate occupied a phylogenetically isolated position (minimum pairwise distance of 1629 core-genome SNPs to its nearest relative). Collectively, these findings highlight the value of integrating WGS into routine One Health surveillance frameworks spanning the food-animal-human interface across South Africa and the wider continent and identify the long-read sequencing of the beef-derived isolate’s plasmid architecture, together with dedicated clinical ExPEC reference collections for serotype-matched comparison, as a priority for follow-up work.

## Figures and Tables

**Table 1:** Antimicrobial resistance determinants identified in the beef-derived isolate.

Gene	Class/Mechanism	Intrinsic/Acquired	Resistance Conferred	Notes
*ampH* (*E. coli* AP012030)	β-Lactamase-like enzyme	Intrinsic	Minimal contribution to β-lactam resistance	Reported as chromosomal in the literature; weak, not a clinical determinant on its own [55].
*ampC1* (*E. coli* FN649414)	AmpC β-Lactamase	Intrinsic	Resistance to penicillins; inducible cephalosporin resistance if overexpressed	Reported as chromosomal in the literature; relevant only with promoter/attenuator mutations [56].
PBP (*E. coli* CP002291)	Target modification	Intrinsic	None unless mutated	Native PBP; β-lactam target; mutations required for resistance [56].
*aadA1*	ANT($3^{\prime \prime }$)-Ia nucleotidyltransferase	Acquired	Streptomycin and spectinomycin	Class 1 integron-associated; no activity against gentamicin, tobramycin, or amikacin; co-located with other cassettes on MGEs [57,58].
*blaEC-15* (NG_049081.1)	β-lactamase	Intrinsic	Aminopenicillin resistance	Reported as chromosomal in the literature; narrow-spectrum, no ESBL activity [59].
*tet*(B) (NG_048163.1)	Tetracycline efflux pump	Acquired	Tetracycline and doxycycline resistance	Commonly reported as plasmid-borne in the literature [60]; localisation not confirmed (short-read assembly only).
*sat2* family (NG_048068.1)	Streptothricin acetyltransferase	Acquired	Streptothricin resistance	Rare but relevant in One Health settings [61].
*acrF* (U00096.3)	RND efflux pump	Intrinsic	Low-level multidrug efflux (fluoroquinolones, chloramphenicol, and tetracyclines)	Contributes to intrinsic tolerance; not a primary AMR gene [62].
*emrD* (AP009048.1)	MFS efflux pump	Intrinsic	Low-level resistance to multiple classes	Background efflux; minor impact unless overexpressed [63].

**Table 2:** Quality of de-novo assembled genomes (397 comparative NCBI SRA genomes plus the beef-derived isolate; N = 398 total).

Quality Metric	Observed Range	Median
Estimated coverage	30–648	53.3
GC content (%)	50–51	50.7
Number of scaffolds	48–457	178
N50	28,515–397,466	90,550
Longest contig	85,613–1,206,009	269,033
Genome size (in Mbps)	4.52–5.57	5.04

**Table 3:** Virulence gene categories identified in the beef-derived isolate.

Category	Genes observed	Key function
Curli (biofilm)	*csgA–G*	Amyloid fibres for biofilm and adhesion.
Type 1 fimbriae	*fimA–I*	Mannose-sensitive adhesion; *FimH* tip adhesin.
*E. coli* common pilus	*ecpRABCDE*	Epithelial adherence; UPEC colonisation support.
Motility/chemotaxis	*flgA–L/N*, *flhA–E*, *fliA/E–T/Y/Z*, *flk*, *cheA/B/R/W/Y/Z*, *and tar*	Flagellum assembly and chemotaxis regulation.
Iron acquisition	*entA–F/S*, *fepA–G*, *and fes*	Enterobactin siderophore system.
Toxin	*hlyE* (ClyA)	Pore-forming cytolysin.
Invasion	*ibeB and ibeC*	Brain endothelial invasion in NMEC models.
T3SS-related (non-LEE)	*espX1/4/5 and espL1/3/4*	Non-LEE effectors in A/E pathogens (not pathotype-defining alone).

**Table 4:** Summary of the pan-resistome across 398 African *E. coli* genomes, grouped by antimicrobial class. Prevalence indicates the number of genomes (out of 398) carrying at least one gene in each category. Genes detected in the beef-derived isolate are indicated with an asterisk (*).

Drug Class	Representative Genes	No. of Unique Genes	Most Prevalent Gene(s) (*n*/398; %)
β-Lactam (intrinsic)	*ampC1* *, *ampH* *, PBP *, and *bla*_EC_ *	11	PBP (398; 100), *ampH* (398; 100), *ampC1* (326/398; 81.9%), and *bla*_EC-15_ * (229; 57.5)
β-Lactam (acquired)	*bla*_TEM_, *bla*_CTX-M_, *bla*_NDM_, *bla*_OXA_, and *bla*_CMY_	27	*bla*_TEM-1_ (238; 59.8), *bla*_CTX-M-15_ (235; 59.0), and *bla*_NDM-5_ (197; 49.5)
Aminoglycoside	*aac(3)-IId*, *aadA1* *, *aadA2*, *aadA5*, *aph($3^{\prime \prime }$)-Ib*, *aph(6)-Id*, and *rmtB1*	12	*aph(6)-Id* (234; 58.8) and *aph($3^{\prime \prime }$)-Ib* (229; 57.5)
Tetracycline	*tet*(B) *, *tet*(A), *tetR*, and *tet*(D)	4	*tet*(B) (191; 48.0), *tet*(A) (156; 39.2), and *tet*R (150; 37.7)
Sulfonamide	*sul1* and *sul2*	3	*sul2* (241; 60.6) and *sul1* (188; 47.2)
Trimethoprim	*dfrA1*, *dfrA12*, *dfrA14*, *dfrA17*, *dfrA4–8*, and *dfrG*	10	*dfrA1* (106; 26.6) and *dfrA12* (95; 23.9)
Efflux pump (intrinsic)	*acrF* *, *emrD* *, *mdtM*, and *emrE*	4	*emrD* (397; 99.7), *acrF* (390; 98.0)
Phenicol	*catA1*, *catB3*, *catB4*, *cmlA6*, and *floR*	7	*catB4* (110; 27.6), *catA1* (55; 13.8), and *floR* (20; 5.0)
Macrolide	*mph(A)*, *mph(B)*, *erm(B)*, *estT*, and *estX-3*	5	*mph(A)* (211; 53.0) and *erm(B)* (53; 13.3)
Quinolone/ Fluoroquinolone	*qnrS1*, *qnrB1*, *qepA*, and *aac($6^{\prime }$)-Ib-cr*	8	*aac($6^{\prime }$)-Ib-cr* (115; 28.9) and *qnrS1* (44; 11.1)
Streptothricin	*sat2* *	1	*sat2* (84; 21.1)
Colistin	*mcr-1.1*	1	*mcr-1.1* (3; 0.8)
Rifamycin	*arr-2*	1	*arr-2* (1; 0.3)
Bleomycin	*ble-MBL*	1	*ble-MBL* (208; 52.3)
Quaternary ammonium	*qacEdelta1* and *qacL*	2	*qacEdelta1* (207; 52.0)
Total		97	

**Table 5:** Summary of plasmid replicon types identified across 398 African *E. coli* genomes, grouped by incompatibility family. Prevalence indicates the number of genomes carrying each replicon type. Replicons detected in the beef-derived isolate are indicated with an asterisk (*).

Replicon Family	Replicon Types Detected	No. of Types	Most Prevalent Type(s) (*n*/398; %)
IncF	IncFIA, IncFIA(HI1), IncFIB(AP001918), IncFIB(H89-PhagePlasmid), IncFIB(K), IncFIB(pB171), IncFIB(pLF82-PhagePlasmid), IncFIB(pQil), IncFIC(FII), IncFII, IncFII(29), IncFII(K), IncFII(pAMA1167-NDM-5), IncFII(pCoo), IncFII(pEH01), IncFII(pHN7A8), IncFII(pRSB107), and IncFII(pSE11)	18	IncFIB(AP001918) (199; 50.0), IncFIA (192; 48.2), and IncFII(29) (112; 28.1)
Col	Col(BS512), Col(IRGK), Col(MG828), Col(MP18), Col(pHAD28), Col156, Col440II, Col440I_1 *, Col8282, ColRNAI, and ColpEC648	11	Col(BS512) (182; 45.7), Col156 (157; 39.4), and Col(pHAD28) (121; 30.4)
IncI	IncI(Gamma), IncI1-I(Alpha), IncI2(Delta), and IncI2	4	IncI1-I(Alpha) (127; 31.9)
IncX	IncX1, IncX3, IncX4, and IncX4_2	4	IncX3 (115; 28.9)
IncB/O/K/Z	IncB/O/K/Z_1, IncB/O/K/Z_2, and IncB/O/K/Z_4	3	IncB/O/K/Z_1 (13; 3.3)
IncHI	IncHI1A, IncHI1B(R27), IncHI1B(pNDM-CIT), IncHI2A, and IncHI2	5	IncHI2 (8; 2.0) and IncHI2A (8; 2.0)
IncY	IncY	1	IncY (26; 6.5)
IncA	IncA	1	IncA (1; 0.3)
IncL	IncL	1	IncL (1; 0.3)
IncN	IncN	1	IncN (1; 0.3)
IncQ	IncQ1	1	IncQ1 (1; 0.3)
p0111	p0111	1	p0111 (14; 3.5)
**Total**		**51**	

**Table 6:** Gene clusters unique to the beef-derived *E. coli* isolate identified from pan-genome analysis.

Gene Cluster	Annotation	*N* in Subset	*N* Total genomes
group_9855	Hypothetical protein	1	1
group_9854	Hypothetical protein	1	1
group_9852	Hypothetical protein	1	1
group_9851	Hypothetical protein	1	1
group_9849	Hypothetical protein	1	1
group_9848	Hypothetical protein	1	1
group_9847	Type II methyltransferase M1.BsuMI	1	1
group_4681	Hypothetical protein	1	1
group_494	Hypothetical protein	1	1

**Table 7:** Core-genome metrics.

Dataset Metric	Value	Interpretation
Total genomes	399 ^1^	Large population study
Total SNPs identified	1,506,494	High-resolution analysis
Recombination blocks	25,706	Extensive HGT detected
Recombination removed	57.7 Mbp	Phylogenetic signal cleaned
Pairwise distance range	0–73,565 SNPs	Extreme genetic diversity
Population median distance	9181 SNPs	Typical relatedness
EM-5_S15 closest relative	1629 SNPs	Genetically distinct
EM-5_S15 median distance	2412 SNPs	Moderately divergent

^1^ The total of 399 includes the K-12 MG1655 genome, used as the reference for read mapping and phylogenetic rooting; it is distinct from the 398 bacterial genomes analysed in this study.

## Data Availability

The original whole-genome sequencing reads presented in this study are openly available in the NCBI Sequence Read Archive (SRA) at https://www.ncbi.nlm.nih.gov/bioproject/PRJNA1483073, accessed on 17 August 2026 (BioProject PRJNA1483073). The *Escherichia coli* isolate EM-5_S15 is deposited under BioSample SAMN61142051 and SRA run SRR39356516 (https://www.ncbi.nlm.nih.gov/sra/SRR39356516, accessed on 17 August 2026). Additional processed data supporting the findings of this study are contained in the article and Supplementary Materials.

## Supplementary Materials

The following supporting information can be downloaded at: https://www.mdpi.com/article/10.3390/microorganisms14092003/s1, Table S1: Antimicrobial resistance determinants identified in the beef-derived *Escherichia coli* isolate (EM-5_S15), including drug class/mechanism and intrinsic vs. acquired status; Table S2: Genomic, taxonomic, AMR gene hit, and plasmid replicon summary for the beef-derived *E. coli* isolate (EM-5_S15); Table S3: Full virulence factor gene inventory identified in the beef-derived *E. coli* isolate (EM-5_S15); Table S4: Virulence gene categories and key functions identified in the beef-derived *E. coli* isolate (EM-5_S15); Table S5: Prevalence of antimicrobial resistance genes among the 398 comparative African *E. coli* genomes; Table S6: Prevalence of plasmid replicons among the 398 comparative African *E. coli* genomes; Table S7: Genomic, MLST, antimicrobial resistance, hypervirulence, extended virulence gene (*fimA–I*, *csgA–G*, *ecpA–E/R*, *entA–F/S*, *fepA–G*, *fes*, *ibeB*, *ibeC*, and *hlyE*), plasmid replicon, and serotyping (ECTyper v1.0) data for the 398 comparative African *E. coli* genomes; Table S8: Distribution of the 398 African *E. coli* genomes by country and isolation source/type; Table S9: Per-sample-source-category prevalence of extended virulence genes (*fimH*, curli operons *csgA–G*, *ecpR*, enterobactin genes *entA/fepA/fes*, invasion genes *ibeB/ibeC*, and *hlyE*) among the 398 comparative African *E. coli* genomes (data supporting Figure 3).

## Abbreviations

The following abbreviations are used in this manuscript:

WGS	Whole-Genome Sequencing
AMR	Antimicrobial Resistance
MALDI-TOF MS	Matrix-Assisted Laser Desorption Ionisation Time-Of-Flight Mass Spectrometry
NCBI	National Centre for Biotechnology Information
SRA	Sequence Reads Archive
ESBL	Extended-Spectrum β-lactamase
WHO	World Health Organisation
CGE	Centre for Genomic Epidemiology
SNP	Single-Nucleotide Polymorphism
HPC	High-Performance Computing
iTOL	Interactive Tree Of Life
ST	Sequence Type
DZ	Algeria
EGY	Egypt
ET	Ethiopia
NE	Niger
ML	Mali
NG	Nigeria
KE	Kenya
TZ	Tanzania
TN	Tunisia
GH	Ghana
ZA	South Africa
ZM	Zambia

## References

[1] Basavaraju M., Gunashree B., Starčič Erjavec M. (2022). *Escherichia coli*: An Overview of Main Characteristics. Escherichia coli—Old and New Insights.

[2] Puvača N., de Llanos Frutos R. (2021). Antimicrobial resistance in *Escherichia coli* strains isolated from humans and pet animals. Antibiotics.

[3] Alhadlaq M.A., Aljurayyad O.I., Almansour A., Al-Akeel S.I., Alzahrani K.O., Alsalman S.A., Yahya R., Al-Hindi R.R., Hakami M.A., Alshahrani S.D. (2024). Overview of pathogenic *Escherichia coli*, with a focus on Shiga toxin-producing serotypes, global outbreaks (1982–2024) and food safety criteria. Gut Pathog..

[4] Sarowska J., Futoma-Koloch B., Jama-Kmiecik A., Frej-Madrzak M., Ksiazczyk M., Bugla-Ploskonska G., Choroszy-Krol I. (2019). Virulence factors, prevalence and potential transmission of extraintestinal pathogenic *Escherichia coli* isolated from different sources: Recent reports. Gut Pathog..

[5] Pakbin B., Brück W.M., Rossen J.W. (2021). Virulence factors of enteric pathogenic *Escherichia coli*: A review. Int. J. Mol. Sci..

[6] Riley L.W. (2020). Distinguishing pathovars from nonpathovars: *Escherichia coli*. Microbiol. Spectr..

[7] Xue M., Li Z., Zhang P., Lei W. (2024). Genomic characteristics of ETT2 gene clusters in avian pathogenic *Escherichia coli* identified by whole-genome sequencing. Pak. Vet. J..

[8] Gebisa E.S., Gerasu M.A., Leggese D.T. (2019). A review on virulence factors of *Escherichia coli*. Anim. Vet. Sci..

[9] Antimicrobial Resistance Collaborators (2022). Global burden of bacterial antimicrobial resistance in 2019: A systematic analysis. Lancet.

[10] World Health Organization Antimicrobial Resistance (WHO Fact Sheet). https://www.who.int/news-room/fact-sheets/detail/antimicrobial-resistance.

[11] Moyane J., Jideani A., Aiyegoro O. (2013). Antibiotics usage in food-producing animals in South Africa and impact on human: Antibiotic resistance. Afr. J. Microbiol. Res..

[12] Sadi M., Akkou M., Martínez-Álvarez S., Carvalho I., Fernández-Fernández R., Abdullahi I.N., Hakem A., Menoueri M.N., Torres C. (2024). Antimicrobial resistance of *Escherichia coli* involved in Algerian bovine carriage, ESBL detection, integron characterization and genetic lineages. Kafkas Univ. Vet. Fak. Derg..

[13] Kizil S., Aydin F.E., Önel A.U., Yildirim M., Güneri̇ C.Ö., Cecen E.M. (2024). Determination of subtypes, serogroups, and serotypes, virulence, and/or toxigenic properties of *Escherichia coli* isolated from cattle, sheep, and goat feces by multiplex PCR. Kafkas Univ. Vet. Fak. Derg..

[14] Bui M.T., Nguyen T.T., Nguyen H.C., Ly K.L., Nguyen T.K. (2024). Antibiotic resistance and pathogenicity of *Escherichia coli* isolated from cattle raised in households in the Mekong Delta, Vietnam. Int. J. Vet. Sci..

[15] Van den Honert M., Gouws P., Hoffman L. (2018). Importance and implications of antibiotic resistance development in livestock and wildlife farming in South Africa: A Review. S. Afr. J. Anim. Sci..

[16] Landers T.F., Cohen B., Wittum T.E., Larson E.L. (2012). A review of antibiotic use in food animals: Perspective, policy, and potential. Public Health Rep..

[17] Naidoo N., Zishiri O.T. (2025). Presence, Pathogenicity, Antibiotic Resistance, and Virulence Factors of *Escherichia coli*: A Review. Bacteria.

[18] Shahrivar E., Rahimi E., Khamesipour F. (2025). Prevalence, identification of virulence genes, and antibiotic resistance properties of Shiga-toxin producing *Escherichia coli* (STEC) strains isolated from ice cream and juice in sales centers. BMC Infect. Dis..

[19] Blair J.M., Webber M.A., Baylay A.J., Ogbolu D.O., Piddock L.J. (2015). Molecular mechanisms of antibiotic resistance. Nat. Rev. Microbiol..

[20] Von Wintersdorff C.J., Penders J., Van Niekerk J.M., Mills N.D., Majumder S., Van Alphen L.B., Savelkoul P.H., Wolffs P.F. (2016). Dissemination of antimicrobial resistance in microbial ecosystems through horizontal gene transfer. Front. Microbiol..

[21] Wu Y., Wang C., Li X., Li F., Jiang M., Liu Z., Liu G., Li J. (2024). Characteristics of the plasmid-mediated colistin-resistance gene *mcr-1* in *Escherichia coli* isolated from a pig farm in Jiangxi. Pak. Vet. J..

[22] Li H., Lin H., Fu Q., Fu Y., Tan J., Wang Y., Zhou R., Sun Z., Li J. (2025). Co-transfer of *mcr-1* and *mcr-3* variant in *Escherichia coli* ST1632 isolate in China: Silence is not always golden. Pak. Vet. J..

[23] Sekwadi P., Ravhuhali K., Mosam A., Essel V., Ntshoe G.M., Shonhiwa A., McCarthy K., Mans J., Taylor M.B., Page N.A. (2018). Waterborne outbreak of gastroenteritis on the KwaZulu-natal coast, South Africa, December 2016/January 2017. Epidemiol. Infect..

[24] Lupindua A.M. (2018). Epidemiology of Shiga toxin-producing *Escherichia coli* O157:H7 in Africa in review. S. Afr. J. Infect. Dis..

[25] Karama M., Mainga A.O., Cenci-Goga B.T., Malahlela M., El-Ashram S., Kalake A. (2019). Molecular profiling and antimicrobial resistance of Shiga toxin-producing *Escherichia coli* O26, O45, O103, O121, O145 and O157 isolates from cattle on cow-calf operations in South Africa. Sci. Rep..

[26] Onyeka L.O., Adesiyun A.A., Keddy K.H., Hassim A., Smith A.M., Thompson P.N. (2022). Characterisation and epidemiological subtyping of Shiga toxin-producing *Escherichia coli* isolated from the beef production chain in Gauteng, South Africa. Prev. Vet. Med..

[27] Madoroba E., Malokotsa K.P., Ngwane C., Lebelo S., Magwedere K. (2022). Presence and virulence characteristics of Shiga toxin *Escherichia coli* and non-shiga toxin–producing *Escherichia coli* O157 in products from animal protein supply chain enterprises in South Africa. Foodborne Pathog. Dis..

[28] Jaja I.F., Oguttu J., Jaja C.J.I., Green E. (2020). Prevalence and distribution of antimicrobial resistance determinants of *Escherichia coli* isolates obtained from meat in South Africa. PLoS ONE.

[29] Onyeka L.O., Adesiyun A.A., Keddy K.H., Madoroba E., Manqele A., Thompson P.N. (2020). Shiga toxin–producing *Escherichia coli* contamination of raw beef and beef-based ready-to-eat products at retail outlets in Pretoria, South Africa. J. Food Prot..

[30] Onyeka L.O., Adesiyun A.A., Keddy K.H., Manqele A., Madoroba E., Thompson P.N. (2021). Prevalence, risk factors and molecular characteristics of Shiga toxin-producing *Escherichia coli* in beef abattoirs in Gauteng, South Africa. Food Control..

[31] Strasheim W., Lowe M., Smith A.M., Etter E.M., Perovic O. (2024). Whole-genome sequencing of human and porcine *Escherichia coli* isolates on a commercial pig farm in South Africa. Antibiotics.

[32] Abdalla S.E., Bester L.A., Abia A.L., Allam M., Ismail A., Essack S.Y., Amoako D.G. (2025). Genomic insights of Antibiotic-Resistant *Escherichia coli* isolated from intensive pig farming in South Africa using ‘Farm-to-Fork’Approach. Antibiotics.

[33] Mkhize L., Marimani M., Duze S.T. (2025). Molecular Characterisation of *Escherichia coli* Collected From an Urban River in Johannesburg, South Africa. Environ. Microbiol. Rep..

[34] Mikhail A., Jenkins C., Dallman T., Inns T., Douglas A., Martín A., Fox A., Cleary P., Elson R., Hawker J. (2018). An outbreak of Shiga toxin-producing *Escherichia coli* O157:H7 associated with contaminated salad leaves: Epidemiological, genomic and food trace back investigations. Epidemiol. Infect..

[35] Cameron M., Perry J., Middleton J., Chaffer M., Lewis J., Keefe G. (2018). Evaluation of MALDI-TOF mass spectrometry and a custom reference spectra expanded database for the identification of bovine-associated coagulase-negative staphylococci. J. Dairy Sci..

[36] Corporation Z.R. Quick-DNA Fungal/Bacterial Miniprep Kit, Catalog No. D6005. https://files.zymoresearch.com/protocols/_d6005_quick-dna_fungal-bacterial_miniprep_kit.pdf.

[37] Biolabs N.E. NEBNext Ultra II FS DNA Library Prep Kit for Illumina, Instruction Manual Version 4.0_7/23, Catalog No. E7805S/L. https://www.neb.com/en/-/media/nebus/files/manuals/manuale6177-e7805.pdf.

[38] Sciences B.C.L. AMPure XP Instructions for Use, Document No. B37419AB. https://www.beckman.com/reagents/genomic/cleanup-and-size-selection/pcr/ampure-xp-protocol.

[39] Pacific Biosciences of California, Inc Onso Sequencing Reagent Pack (300 Cycles), Document No. 102-974-500, Rev. 04. https://www.pacb.com/wp-content/uploads/Insert-Onso-sequencing-reagent-pack-300-cycles.pdf.

[40] NCBI SRA Toolkit (Sratools) Version 3.2.0, National Center for Biotechnology Information. Public Domain. United States Government Work. https://github.com/ncbi/sra-tools.

[41] CDCgov CDCgov/Phoenix: v2.3.1 (v2.3.1), 2023. https://zenodo.org/records/8147510.

[42] Bushnell B. BBMap: A Fast, Accurate, Splice-Aware Aligner. https://sourceforge.net/projects/bbmap/.

[43] Chen S., Zhou Y., Chen Y., Gu J. (2018). fastp: An ultra-fast all-in-one FASTQ preprocessor. Bioinformatics.

[44] Wood D.E., Lu J., Langmead B. (2019). Improved Metagenomic Analysis with Kraken 2. Genome Biol..

[45] Prjibelski A., Antipov D., Meleshko D., Lapidus A., Korobeynikov A. (2020). Using SPAdes de-novo assembler. Curr. Protoc. Bioinform..

[46] Bankevich A., Nurk S., Antipov D., Gurevich A.A., Dvorkin M., Kulikov A.S., Lesin V.M., Nikolenko S.I., Pham S., Prjibelski A.D. (2012). SPAdes: A new genome assembly algorithm and its applications to single-cell sequencing. J. Comput. Biol..

[47] Gurevich A., Saveliev V., Vyahhi N., Tesler G. (2013). QUAST: Quality assessment tool for genome assemblies. Bioinformatics.

[48] Seemann T. (2014). Prokka: Rapid prokaryotic genome annotation. Bioinformatics.

[49] Feldgarden M., Brover V., Gonzalez-Escalona N., Frye J.G., Haendiges J., Haft D.H., Hoffmann M., Pettengill J.B., Prasad A.B., Tillman G.E. (2021). AMRFinderPlus and the Reference Gene Catalog facilitate examination of the genomic links among antimicrobial resistance, stress response, and virulence. Sci. Rep..

[50] Inouye M., Dashnow H., Raven L.A., Schultz M.B., Pope B.J., Tomita T., Zobel J., Holt K.E. (2014). SRST2: Rapid genomic surveillance for public health and hospital microbiology labs. Genome Med..

[51] Stanton R.A., Vlachos N., Halpin A.L. (2022). GAMMA: A tool for the rapid identification, classification and annotation of translated gene matches from sequencing data. Bioinformatics.

[52] Bortolaia V., Kaas R.S., Ruppe E., Roberts M.C., Schwarz S., Cattoir V., Philippon A., Allesoe R.L., Rebelo A.R., Florensa A.F. (2020). ResFinder 4.0 for predictions of phenotypes from genotypes. J. Antimicrob. Chemother..

[53] Gupta S.K., Padmanabhan B.R., Diene S.M., Lopez-Rojas R., Kempf M., Landraud L., Rolain J.M. (2014). ARG-ANNOT, a new bioinformatic tool to discover antibiotic resistance genes in bacterial genomes. Antimicrob. Agents Chemother..

[54] Robertson J., Nash J.H. (2018). MOB-suite: Software tools for clustering, reconstruction and typing of plasmids from draft assemblies. Microb. Genom..

[55] Henderson T.A., Young K.D., Denome S.A., Elf P.K. (1997). AmpC and AmpH, proteins related to the class C beta-lactamases, bind penicillin and contribute to the normal morphology of *Escherichia coli*. J. Bacteriol..

[56] Jacoby G.A. (2009). AmpC *β*-lactamases. Clin. Microbiol. Rev..

[57] Partridge S.R., Kwong S.M., Firth N., Jensen S.O. (2018). Mobile genetic elements associated with antimicrobial resistance. Clin. Microbiol. Rev..

[58] Lu H., Zhao W.J., Hu Y.Q., Shen W., Chen Y.F., Wei Z.Q. (2013). Identification and Characterization of Integron-Mediated Antibiotic Resistance in the Phytopathogen *Xanthomonas oryzae* pv. *oryzae*. PLoS ONE.

[59] Schmidt J., Zdarska V., Kolar M., Mlynarcik P. (2023). Analysis of BlaEC family class C beta-lactamase. FEMS Microbiol. Lett..

[60] Chopra I., Roberts M. (2001). Tetracycline antibiotics: Mode of action, applications, molecular biology, and epidemiology of bacterial resistance. Microbiol. Mol. Biol. Rev..

[61] Tietze E., Brevet J. (1990). Nucleotide sequence of the streptothricin-acetyl-transferase gene *sat-2*. Nucleic Acids Res..

[62] Jellen-Ritter A.S., Kern W.V. (2001). Enhanced Expression of the Multidrug Efflux Pumps AcrAB and AcrEF Associated with Insertion Element Transposition in *Escherichia coli* Mutants Selected with a Fluoroquinolone. Antimicrob. Agents Chemother..

[63] Yin Y., He X., Szewczyk P., Nguyen T., Chang G. (2006). Structure of the multidrug transporter *EmrD* from *Escherichia coli*. Science.

[64] Seemann T. ABRicate. v1.2.0. https://github.com/tseemann/abricate.

[65] Chen L., Zheng D., Liu B., Yang J., Jin Q. (2016). VFDB 2016: Hierarchical and refined dataset for big data analysis—10 years on. Nucleic Acids Res..

[66] Le K., Laing C. ecoli_vf: Curated Virulence Factors for *Escherichia coli*. https://github.com/phac-nml/ecoli_vf.

[67] Joensen K.G., Scheutz F., Lund O., Hasman H., Kaas R.S., Nielsen E.M., Aarestrup F.M. (2014). Real-time whole-genome sequencing for routine typing, surveillance, and outbreak detection of verotoxigenic *Escherichia coli*. J. Clin. Microbiol..

[68] Seemann T. mlst: Scan Contig Files Against PubMLST Typing Schemes. https://github.com/tseemann/mlst.

[69] Jolley K.A., Maiden M.C. (2010). BIGSdb: Scalable analysis of bacterial genome variation at the population level. BMC Bioinform..

[70] Zhou Z., Charlesworth J., Achtman M. (2021). HierCC: A multi-level clustering scheme for population assignments based on core genome MLST. Bioinformatics.

[71] Joensen K.G., Tetzschner A.M., Iguchi A., Aarestrup F.M., Scheutz F. (2015). Rapid and easy in silico serotyping of *Escherichia coli* isolates by use of whole-genome sequencing data. J. Clin. Microbiol..

[72] Roer L., Johannesen T.B., Hansen F., Stegger M., Tchesnokova V., Sokurenko E., Garibay N., Allesøe R., Thomsen M.C., Lund O. (2018). CHTyper, a web tool for subtyping of extraintestinal pathogenic *Escherichia coli* based on the fumC and fimH alleles. J. Clin. Microbiol..

[73] Bessonov K., Laing C., Robertson J., Yong I., Ziebell K., Gannon V.P., Nichani A., Arya G., Nash J.H., Christianson S. (2021). ECTyper: In silico *Escherichia coli* serotype and species prediction from raw and assembled whole-genome sequence data. Microb. Genom..

[74] Carattoli A., Zankari E., García-Fernández A., Voldby Larsen M., Lund O., Villa L., Møller Aarestrup F., Hasman H. (2014). In silico detection and typing of plasmids using PlasmidFinder and plasmid multilocus sequence typing. Antimicrob. Agents Chemother..

[75] Tonkin-Hill G., MacAlasdair N., Ruis C., Weimann A., Horesh G., Lees J.A., Gladstone R.A., Lo S., Beaudoin C., Floto R.A. (2020). Producing polished prokaryotic pangenomes with the Panaroo pipeline. Genome Biol..

[76] Tettelin H., Masignani V., Cieslewicz M.J., Donati C., Medini D., Ward N.L., Angiuoli S.V., Crabtree J., Jones A.L., Durkin A.S. (2005). Genome analysis of multiple pathogenic isolates of *Streptococcus agalactiae*: Implications for the microbial “pan-genome”. Proc. Natl. Acad. Sci. USA.

[77] Waskom M.L. (2024). Seaborn: Statistical Data Visualization.

[78] Seemann T. Snippy: Rapid Haploid Variant Calling and Core Genome Alignment. v4.6.0. https://github.com/tseemann/snippy.

[79] Croucher N.J., Page A.J., Connor T.R., Delaney A.J., Keane J.A., Bentley S.D., Parkhill J., Harris S.R. (2015). Rapid phylogenetic analysis of large samples of recombinant bacterial whole-genome sequences using Gubbins. Nucleic Acids Res..

[80] Letunic I., Bork P. (2024). Interactive Tree of Life (iTOL) v6: Recent updates to the phylogenetic tree display and annotation tool. Nucleic Acids Res..

[81] Seemann T. snp-dists: Pairwise SNP Distance Matrix from a FASTA Alignment. v0.8.2. https://github.com/tseemann/snp-dists.

[82] Dallman T.J., Ashton P.M., Byrne L., Perry N.T., Petrovska L., Ellis R., Allison L., Hanson M., Holmes A., Gunn G.J. (2015). Applying phylogenomics to understand the emergence of Shiga-toxin-producing *Escherichia coli* O157:H7 strains causing severe human disease in the UK. Microb. Genom..

[83] Raro O.H.F., Le Terrier C., Nordmann P., Poirel L. (2024). Impact of extended-spectrum chromosomal AmpC (ESAC) of *Escherichia coli* on susceptibility to cefiderocol. Microbiol. Spectr..

[84] Totté J.E., Quiblier C., Nolte O., Hinic V., Wunderink H.F., Egli A., Mancini S. (2025). Phenotypic susceptibility profiles of AmpC-and/or extended-spectrum beta-lactamase-(co) producing *Escherichia coli* strains. JAC-Antimicrob. Resist..

[85] Reingold J., Starr N., Maurer J., Lee M.D. (1999). Identification of a new *Escherichia coli* She haemolysin homolog in avian *E. coli*. Vet. Microbiol..

[86] Linkevicius M., Bonnin R.A., Alm E., Svartström O., Apfalter P., Hartl R., Hasman H., Roer L., Räisänen K., Dortet L. (2023). Rapid cross-border emergence of NDM-5-producing *Escherichia coli* in the European Union/European Economic Area, 2012 to June 2022. Eurosurveillance.

[87] Xia C., Yan R., Liu C., Zhai J., Zheng J., Chen W., Cao X. (2024). Epidemiological and genomic characteristics of global bla NDM-carrying *Escherichia coli*. Ann. Clin. Microbiol. Antimicrob..

[88] ECDC E., EMA (2024). Antimicrobial consumption and resistance in bacteria from humans and food-producing animals. EFSA J..

[89] Ma T., Zaheer R., McAllister T.A., Guo W., Li F., Tu Y., Diao Q., Guan L.L. (2022). Expressions of resistome is linked to the key functions and stability of active rumen microbiome. Anim. Microbiome.

[90] Franck E., Crofts T.S. (2024). History of the streptothricin antibiotics and evidence for the neglect of the streptothricin resistome. npj Antimicrob. Resist..

[91] Ribeiro L.F., Nespolo N.M., Rossi G.A.M., Fairbrother J.M. (2024). Exploring extended-spectrum beta-lactamase (ESBL)-producing *Escherichia coli* in food-producing animals and animal-derived foods. Pathogens.

[92] Ramírez-Castillo F.Y., Guerrero-Barrera A.L., Avelar-González F.J. (2023). An overview of carbapenem-resistant organisms from food-producing animals, seafood, aquaculture, companion animals, and wildlife. Front. Vet. Sci..

[93] Tursi S.A., Tükel Ç. (2018). Curli-containing enteric biofilms inside and out: Matrix composition, immune recognition, and disease implications. Microbiol. Mol. Biol. Rev..

[94] Munhoz D.D., Richards A.C., Santos F.F., Mulvey M.A., Piazza R.M.F. (2023). *E. coli* Common pili promote the fitness and virulence of a hybrid aEPEC/ExPEC strain within diverse host environments. Gut Microbes.

[95] Sora V.M., Meroni G., Martino P.A., Soggiu A., Bonizzi L., Zecconi A. (2021). Extraintestinal pathogenic *Escherichia coli*: Virulence factors and antibiotic resistance. Pathogens.

[96] Qiu L., Chirman D., Clark J.R., Xing Y., Hernandez Santos H., Vaughan E.E., Maresso A.W. (2024). Vaccines against extraintestinal pathogenic *Escherichia coli* (ExPEC): Progress and challenges. Gut Microbes.

[97] Huang S.H., Chen Y.H., Fu Q., Stins M., Wang Y., Wass C., Kim K.S. (1999). Identification and characterization of an *Escherichia coli* invasion gene locus, *ibeB*, required for penetration of brain microvascular endothelial cells. Infect. Immun..

[98] Kim K.S. (2016). Human meningitis-associated *Escherichia coli*. Ecosal Plus.

[99] Ludwig A., Von Rhein C., Bauer S., Huttinger C., Goebel W. (2004). Molecular analysis of cytolysin A (ClyA) in pathogenic *Escherichia coli* strains. J. Bacteriol..

[100] Murase K. (2022). Cytolysin A (ClyA): A bacterial virulence factor with potential applications in nanopore technology, vaccine development, and tumor therapy. Toxins.

[101] Bugarel M., Martin A., Fach P., Beutin L. (2011). Virulence gene profiling of enterohemorrhagic (EHEC) and enteropathogenic (EPEC) *Escherichia coli* strains: A basis for molecular risk assessment of typical and atypical EPEC strains. BMC Microbiol..

[102] Kaper J.B., Nataro J.P., Mobley H.L. (2004). Pathogenic *Escherichia coli*. Nat. Rev. Microbiol..

[103] Jaureguy F., Landraud L., Passet V., Diancourt L., Frapy E., Guigon G., Carbonnelle E., Lortholary O., Clermont O., Denamur E. (2008). Phylogenetic and genomic diversity of human bacteremic *Escherichia coli* strains. BMC Genom..

[104] Jolley K.A., Bray J.E., Maiden M.C. (2018). Open-access bacterial population genomics: BIGSdb software, the PubMLST. org website and their applications. Wellcome Open Res..

[105] Manges A.R., Smith S.P., Lau B.J., Nuval C.J., Eisenberg J.N., Dietrich P.S., Riley L.W. (2007). Retail meat consumption and the acquisition of antimicrobial resistant *Escherichia coli* causing urinary tract infections: A case–control study. Foodborne Pathog. Dis..

[106] Manges A. (2016). *Escherichia coli* and urinary tract infections: The role of poultry-meat. Clin. Microbiol. Infect..

[107] Moulin-Schouleur M., Schouler C., Tailliez P., Kao M.R., Brée A., Germon P., Oswald E., Mainil J., Blanco M., Blanco J. (2006). Common virulence factors and genetic relationships between O18: K1: H7 *Escherichia coli* isolates of human and avian origin. J. Clin. Microbiol..

[108] Krishnan S., Chang A.C., Hodges J., Couraud P.O., Romero I.A., Weksler B., Nicholson B.A., Nolan L.K., Prasadarao N.V. (2015). Serotype O18 avian pathogenic and neonatal meningitis *Escherichia coli* strains employ similar pathogenic strategies for the onset of meningitis. Virulence.

[109] Arredondo-Alonso S., Willems R.J., Van Schaik W., Schürch A.C. (2017). On the (im) possibility of reconstructing plasmids from whole-genome short-read sequencing data. Microb. Genom..

[110] Hall J.P., Brockhurst M.A., Harrison E. (2017). Sampling the mobile gene pool: Innovation via horizontal gene transfer in bacteria. Philos. Trans. R. Soc. B Biol. Sci..

[111] Olaitan M.O., Orababa O.Q., Shittu R.B., Obunukwu G.M., Kade A.E., Arowolo M.T., Oyediran A.A., Yusuff R.A. (2025). Prevalence of ESBL-producing *Escherichia coli* in sub-Saharan Africa: A meta-analysis using a One Health approach. One Health.

[112] Somda N.S., Nyarkoh R., Kotey F.C., Tetteh-Quarcoo P.B., Donkor E.S. (2024). A systematic review and meta-analysis of carbapenem-resistant *Enterobacteriaceae* in West Africa. BMC Med. Genom..

[113] Khajuria A., Praharaj A.K., Kumar M., Grover N. (2014). Emergence of *Escherichia coli*, co-producing *NDM-1* and *OXA-48* carbapenemases, in urinary isolates, at a tertiary care centre at Central India. J. Clin. Diagn. Res. JCDR.

[114] Liu Y.Y., Wang Y., Walsh T.R., Yi L.X., Zhang R., Spencer J., Doi Y., Tian G., Dong B., Huang X. (2016). Emergence of plasmid-mediated colistin resistance mechanism MCR-1 in animals and human beings in China: A microbiological and molecular biological study. Lancet Infect. Dis..

[115] Villa L., García-Fernández A., Fortini D., Carattoli A. (2010). Replicon sequence typing of IncF plasmids carrying virulence and resistance determinants. J. Antimicrob. Chemother..

[116] McNally A., Kallonen T., Connor C., Abudahab K., Aanensen D.M., Horner C., Peacock S.J., Parkhill J., Croucher N.J., Corander J. (2019). Diversification of colonization factors in a multidrug-resistant *Escherichia coli* lineage evolving under negative frequency-dependent selection. mBio.

[117] Li X., Fu Y., Shen M., Huang D., Du X., Hu Q., Zhou Y., Wang D., Yu Y. (2018). Dissemination of *blaNDM-5* gene via an IncX3-type plasmid among non-clonal *Escherichia coli* in China. Antimicrob. Resist. Infect. Control..

[118] Tian D., Wang B., Zhang H., Pan F., Wang C., Shi Y., Sun Y. (2020). Dissemination of the *blaNDM-5* gene via IncX3-type plasmid among *Enterobacteriaceae* in children. mSphere.

[119] Johnson T.J., Wannemuehler Y., Doetkott C., Johnson S.J., Rosenberger S.C., Nolan L.K. (2007). Plasmid replicon typing of commensal and pathogenic *Escherichia coli* isolates. Appl. Environ. Microbiol..

[120] Reid C.J., DeMaere M.Z., Djordjevic S.P. (2019). Australian porcine clonal complex 10 (CC10) *Escherichia coli* belong to multiple sublineages of a highly diverse global CC10 phylogeny. Microb. Genom..

[121] Kocsis B., Gulyás D., Szabó D. (2022). Emergence and dissemination of extraintestinal pathogenic high-risk international clones of *Escherichia coli*. Life.

[122] Garcia-Fernandez A., Villa L., Bibbolino G., Bressan A., Trancassini M., Pietropaolo V., Venditti M., Antonelli G., Carattoli A. (2020). Novel insights and features of the NDM-5-producing *Escherichia coli* sequence type 167 high-risk clone. mSphere.

[123] Nicolas-Chanoine M.H., Bertrand X., Madec J.Y. (2014). *Escherichia coli* ST131, an intriguing clonal group. Clin. Microbiol. Rev..

[124] Manges A.R., Geum H.M., Guo A., Edens T.J., Fibke C.D., Pitout J.D.D. (2019). Global extraintestinal pathogenic *Escherichia coli* (ExPEC) lineages. Clin. Microbiol. Rev..

[125] Johnson T.J., Elnekave E., Miller E.A., Munoz-Aguayo J., Flores Figueroa C., Johnston B., Nielson D.W., Logue C.M., Johnson J.R. (2019). Phylogenomic analysis of extraintestinal pathogenic *Escherichia coli* sequence type 1193, an emerging multidrug-resistant clonal group. Antimicrob. Agents Chemother..

[126] Pitout J.D., Peirano G., Chen L., DeVinney R., Matsumura Y. (2022). *Escherichia coli* ST1193: Following in the footsteps of *E. coli* ST131. Antimicrob. Agents Chemother..

[127] Alba P., Leekitcharoenphon P., Franco A., Feltrin F., Ianzano A., Caprioli A., Stravino F., Hendriksen R.S., Bortolaia V., Battisti A. (2018). Molecular epidemiology of mcr-encoded colistin resistance in *Enterobacteriaceae* from food-producing animals in Italy revealed through the EU harmonized antimicrobial resistance monitoring. Front. Microbiol..

[128] Adzitey F., Asante J., Kumalo H.M., Khan R.B., Somboro A.M., Amoako D.G. (2020). Genomic investigation into the virulome, pathogenicity, stress response factors, clonal lineages, and phylogenetic relationship of *Escherichia coli* strains isolated from meat sources in Ghana. Genes.

[129] Roy Chowdhury P., Hastak P., DeMaere M., Wyrsch E., Li D., Elankumaran P., Dolejska M., Browning G.F., Marenda M.S., Gottlieb T. (2023). Phylogenomic analysis of a global collection of *Escherichia coli* ST38: Evidence of interspecies and environmental transmission?. mSystems.

[130] Mo S.S., Fiskebeck E.Z., Slettemeås J.S., Lagesen K., Nilsson O., Naseer U., Jørgensen S.B., Thorsteinsdottir T.R., Sunde M. (2023). *Escherichia coli* multilocus sequence type 38 from humans and broiler production represent distinct monophyletic groups. Front. Microbiol..

[131] Rasko D.A., Rosovitz M., Myers G.S., Mongodin E.F., Fricke W.F., Gajer P., Crabtree J., Sebaihia M., Thomson N.R., Chaudhuri R. (2008). The pangenome structure of *Escherichia coli*: Comparative genomic analysis of *E. coli* commensal and pathogenic isolates. J. Bacteriol..

[132] Touchon M., Hoede C., Tenaillon O., Barbe V., Baeriswyl S., Bidet P., Bingen E., Bonacorsi S., Bouchier C., Bouvet O. (2009). Organised genome dynamics in the *Escherichia coli* species results in highly diverse adaptive paths. PLoS Genet..

[133] Wilson G.G., Murray N.E. (1991). Restriction and modification systems. Annu. Rev. Genet..

[134] Oliveira P.H., Touchon M., Rocha E.P.C. (2016). Regulation of genetic flux between bacteria by restriction–modification systems. Proc. Natl. Acad. Sci. USA.

[135] Didelot X., Maiden M.C.J. (2010). Impact of recombination on bacterial evolution. Trends Microbiol..

