# Supplementary material for: Whole-Genome Sequencing of Escherichia coli O18ab:H11 from South African Beef: Antimicrobial Resistance, Virulence, and a Rare Sequence Type
Source: Microorganisms. 2026 Sep 9;14(9):2003. doi: 10.3390/microorganisms14092003 (PMC13609402; doi:10.3390/microorganisms14092003)
Supplement: Supplementary file 1 [file microorganisms-14-02003-s001.zip › Table S1.pdf]

Table S1: Antimicrobial resistance determinants identified in the beef-derived *Escherichia coli* isolate (EM-5_S15), including drug class/mechanism and intrinsic vs. acquired status.

Gene	Class / Mechanism	Intrinsic / Acquired	Resistance Conferred	Notes
***ampH* (*E. coli* AP012030)**	β-lactamase-like enzyme	Intrinsic	Minimal contribution to β-lactam resistance	Chromosomal; weak activity; not a clinical AMR determinant on its own
***ampC1* (*E. coli* FN649414)**	AmpC β-lactamase	Intrinsic	Resistance to penicillins; inducible cephalosporin resistance if overexpressed	Chromosomal; clinically relevant only with promoter/attenuator mutations
**Penicillin-Binding Protein (*E. coli* CP002291)**	Target modification	Intrinsic	None unless mutated	Native PBP; β-lactam target; mutations required for resistance
***blaEC-15* (NG_049081.1)**	β-lactamase	Intrinsic	Aminopenicillin resistance	Narrow-spectrum β-lactamase; does not confer ESBL activity
***tet(B)* (NG_048163.1)**	Tetracycline efflux pump	**Acquired**	Tetracycline, doxycycline resistance	Common plasmid-borne determinant
aadA1	Aminoglycoside nucleotidyltransferase (*ANT(3'')-Ia*)	**Acquired**	Streptomycin and spectinomycin resistance	Encodes ANT(3'')-Ia; commonly integron-associated (class 1 integrons); does not confer resistance to gentamicin, tobramycin, or

				amikacin; frequently co-located with other resistance cassettes on mobile genetic elements
**sat2 family (NG_048068.1)**	Streptothricin acetyltransferase	**Acquired**	Streptothricin resistance	Rare but relevant in One-Health settings
***acrF* (U00096.3)**	RND efflux pump	Intrinsic	Low-level multidrug efflux (fluoroquinolones, chloramphenicol, tetracyclines)	Contributes to intrinsic tolerance; not a primary AMR gene
***emrD* (AP009048.1)**	MFS efflux pump	Intrinsic	Low-level resistance to multiple classes	
