# Supplementary material for: Whole-Genome Sequencing of Escherichia coli O18ab:H11 from South African Beef: Antimicrobial Resistance, Virulence, and a Rare Sequence Type
Source: Microorganisms. 2026 Sep 9;14(9):2003. doi: 10.3390/microorganisms14092003 (PMC13609402; doi:10.3390/microorganisms14092003)
Supplement: Supplementary file 1 [file microorganisms-14-02003-s001.zip › Table S3.pdf]

Table S3: Full virulence factor gene inventory identified in the beef-derived *E. coli* isolate (EM-5_S15)

GENE	START	END	STRAND	COVERAGE	GAPS	%COVERAGE	%IDENTITY	ACCESSION	PRODUCT
aec30	1382	4214	+	69-2901/2901	0/0	97.66	99.93	gi:386612379	type VI secretion protein IcmFhypothetical protein
aec31	4135	5577	+	1-1443/1443	0/0	100.00	99.86	gi:218693681	hypothetical protein
aec32	5601	6083	+	1-483/483	0/0	100.00	99.79	gi:260866361	Hcp-like protein
artj	61818	62549	-	1-732/732	0/0	100.00	99.73	SPG000096	E.coli artP artI artQ artM and artJ genes.
aslA	14853	16281	+	1-1428/1428	1/1	100.00	93.00	gi:9965749	putative arylsulfatase
b2854	21327	21802	+	1-477/477	1/1	99.79	98.95	SPG000125	Putative lytic transglycosylase Adherence Hemorrhagic coli pilus
cadA	8055	10230	+	1-2176/2176	0/0	100.00	99.27	SPG000027	gb|AY319765.1|:1081-3256 Escherichia coli cadBA operon partial sequence
cheA	22452	24416	-	1-1965/1965	0/0	100.00	98.68	gi:15802300	chemotaxis protein CheA
cheB	16544	17593	-	1-1050/1050	0/0	100.00	98.67	gi:15802295	chemotaxis-specific methylesterase
cheR	17596	18456	-	1-861/861	0/0	100.00	99.30	gi:15802296	chemotaxis methyltransferase CheR
cheW	21928	22431	-	1-504/504	0/0	100.00	98.81	gi:15802299	purine-binding chemotaxis protein
cheY	16140	16529	-	1-390/390	0/0	100.00	99.23	gi:15802294	chemotaxis regulatory protein CheY
cheZ	15485	16129	-	1-645/645	0/0	100.00	99.38	gi:15802293	chemotaxis regulator CheZ
csgA	6866	7321	+	1-459/459	1/3	99.35	93.03	gi:15801159	cryptic curlin major subunit
csgB	6343	6825	+	1-483/483	0/0	100.00	99.79	VFG045792	minor curlin subunit CsgB
csgC	7380	7712	+	1-333/333	0/0	100.00	99.10	gi:15801160	putative autoagglutination protein
csgD	4965	5615	-	1-651/651	0/0	100.00	99.23	VFG045791	putative 2-component transcriptional regulator for 2nd curli operon

csgE	4571	4960	-	1-390/390	0/0	100.00	98.97	gi:15801156	curli assembly protein CsgE
csgF	4130	4546	-	1-417/417	0/0	100.00	99.28	gi:15801155	curli assembly protein CsgF
csgG	3269	4103	-	1-834/834	1/1	100.00	98.08	gi:15801154	curli production assembly/transport component 2nd curli operon
ecpA	3232	3819	+	1-588/588	0/0	100.00	100.00	gi:16128278	conserved protein
ecpB	3828	4545	+	1-717/717	1/1	100.00	97.63	gi:26246301	Hypothetical protein yagY precursor
ecpC	4571	7096	+	1-2526/2526	0/0	100.00	100.00	gi:16128276	predicted aromatic compound dioxygenase
ecpD	7086	8729	+	1-1644/1644	0/0	100.00	100.00	gi:386612485	hypothetical protein
ecpE	8653	9408	+	1-756/756	0/0	100.00	97.49	gi:26246298	Hypothetical protein yagV precursor
ecpR	2567	3157	+	1-591/591	0/0	100.00	99.83	gi:16128279	predicted regulator
ECs3712	21847	22479	-	1-633/633	0/0	100.00	97.63	gi:15832966	hypothetical protein
ECS88_3547	10308	11192	-	1-885/885	0/0	100.00	99.77	gi:218560235	lipoprotein NlpI
ehaB	8999	12001	+	1-2949/2949	7/56	99.97	92.68	gi:222155165	structural protein
entA	6009	6755	+	1-747/747	0/0	100.00	95.98	gi:26246575	23-dihydro-23-dihydroxybenzoate dehydrogenase
entB	5152	6009	+	1-858/858	0/0	100.00	98.37	gi:26246574	isochorismatase
entC	2331	3518	+	1-1188/1188	0/0	100.00	97.73	gi:26246572	isochorismate synthase 1
entD	185	955	-	1-771/771	0/0	100.00	95.20	gi:26246560	phosphopantetheinyl transferase component of enterobactin synthase multienzyme complex
entE	3528	5138	+	1-1611/1611	0/0	100.00	95.59	gi:26246573	23-dihydroxybenzoate-AMP ligase component of enterobactin synthase multienzyme complex
entF	4883	8736	+	1-3854/3882	0/0	99.28	95.64	gi:26246565	enterobactin synthase multienzyme complex component ATP-dependent

entS	1	1008	+	244-1251/1251	0/0	80.58	94.64	gi:26246570	enterobactin exporter iron-regulated
epaO	30394	31381	-	1-987/987	1/1	100.00	96.56	gi:15832980	surface presentation of antigens protein SpaO
epaP	29739	30404	-	1-666/666	0/0	100.00	98.20	gi:15832979	surface presentation of antigens protein SpaP
epaQ	29469	29729	-	1-261/261	0/0	100.00	95.78	gi:15832978	EpaQ
epaR	29017	29480	-	3-466/468	0/0	99.15	96.98	VFG042210	-
epaS	27571	28691	-	1-1122/1122	3/3	99.82	96.79	gi:15832975	surface presentation of antigens protein SpaS
eprH	25277	26007	-	5-735/735	0/0	99.46	98.22	gi:15832973	EprH
eprI	25024	25263	-	1-240/240	0/0	100.00	97.50	gi:15832972	EprI
eprJ	24672	25004	-	1-333/333	0/0	100.00	98.50	gi:15832971	EprJ
eprK	23941	24675	-	1-735/735	0/0	100.00	98.78	gi:15832970	EprK
espL1	26474	28372	-	1-1899/1899	2/2	99.95	97.53	gi:15831681	hypothetical protein
espL3	15354	17231	+	1-1878/1878	0/0	100.00	98.94	SPG000024	gi|47118301:4652971-4654848 Escherichia coli O157:H7 str. Sakai DNA complete genome PTS system arbutin-like IIC component
espL4	3541	5115	-	1-1575/1575	0/0	100.00	99.30	gi:260870723	T3SS effector-like protein EspL
espR1	17628	18958	+	1-1332/1332	1/1	99.92	99.70	gi:260867911	T3SS effector-like protein EspR
espX1	38512	39932	+	1-1422/1422	1/1	99.93	95.22	gi:15799704	Type III secretion system effector EspX1
espX4	33345	34925	+	1-1581/1581	2/2	99.94	94.12	gi:15804631	Type III secretion system effector EspX4
espX5	27918	29210	-	1-1293/1293	0/0	100.00	97.37	gi:291285483	hypothetical protein
etrA	26714	27214	+	1-501/501	0/0	100.00	96.41	gi:15832974	transcriptional regulator
fepA	980	3220	-	1-2241/2241	0/0	100.00	96.74	gi:26246561	ferrienterobactin outer membrane transporter

fepB	1012	1968	-	1-957/957	0/0	100.00	97.49	gi:26246571	ferrienterobactin ABC transporter periplasmic binding protein
fepC	10110	10925	-	1-816/816	0/0	100.00	97.30	gi:26246567	ferrienterobactin ABC transporter ATPase
fepD	11911	12750	-	178-1017/1017	0/0	82.60	95.24	gi:26246569	ferrienterobactin ABC transporter permease
fepE	8980	10113	+	1-1134/1134	0/0	100.00	94.53	gi:26246566	ferric enterobactin transport protein FepE
fepG	10922	11914	-	1-993/993	0/0	100.00	94.06	gi:26246568	iron-enterobactin ABC transporter permease
fes	3463	4665	+	1-1203/1203	0/0	100.00	96.67	gi:26246563	enterobactin/ferric enterobactin esterase
fimA	75	576	-	1-502/549	0/0	91.44	99.80	gi:260871034	major type 1 subunit fimbrin (pilin) FimA
fimB	2131	2733	-	1-603/603	0/0	100.00	100.00	gi:260858480	tyrosine recombinase
fimC	776	1501	+	1-726/726	0/0	100.00	99.72	gi:218707983	chaperone periplasmic
fimD	1568	4204	+	1-2637/2637	0/0	100.00	99.51	gi:260858485	outer membrane usher protein FimD
fimE	1057	1653	-	1-597/597	0/0	100.00	100.00	gi:260858481	tyrosine recombinase
fimF	4214	4744	+	1-531/531	0/0	100.00	100.00	gi:260847134	minor component FimF of type 1 fimbriae
fimF	16647	17177	-	1-531/531	0/0	100.00	99.62	gi:410482684	fimbrial-like adhesin protein
fimG	4757	5260	+	1-504/504	0/0	100.00	100.00	gi:260858487	minor component FimG of type 1 fimbriae
fimG	16131	16634	-	1-504/504	0/0	100.00	99.41	gi:410482685	fimbrial-like adhesin protein
fimH	5280	6182	+	1-903/903	0/0	100.00	99.78	gi:260858488	minor component FimH of type 1 fimbriae
fimI	79	739	+	3-663/663	0/0	99.70	98.79	gi:117626623	FimI fimbrial protein
flgA	28378	29037	+	1-660/660	0/0	100.00	96.97	gi:15801189	flagellar basal body P-ring biosynthesis protein FlgA

flgB	27807	28223	-	1-417/417	0/0	100.00	99.52	gi:15801190	flagellar basal body rod protein FlgB
flgC	27399	27803	-	1-405/405	0/0	100.00	99.51	gi:15801191	flagellar basal body rod protein FlgC
flgD	26692	27387	-	1-696/696	0/0	100.00	99.14	gi:15801192	flagellar basal body rod modification protein
flgE	25459	26667	-	1-1206/1206	1/3	100.00	94.79	gi:15801193	flagellar hook protein FlgE
flgF	24684	25439	-	1-756/756	0/0	100.00	96.56	gi:15801194	flagellar basal body rod protein FlgF
flgG	23730	24512	-	1-783/783	0/0	100.00	99.36	gi:15801195	flagellar basal body rod protein FlgG
flgH	22979	23677	-	1-699/699	0/0	100.00	99.57	gi:15801196	flagellar basal body L-ring protein
flgI	21870	22967	-	1-1098/1098	0/0	100.00	97.54	gi:15801197	flagellar basal body P-ring protein
flgJ	20929	21870	-	1-942/942	0/0	100.00	98.62	gi:15801198	flagellar rod assembly protein/muramidase FlgJ
flgK	19220	20863	-	1-1644/1644	0/0	100.00	98.42	gi:15801199	flagellar hook-associated protein FlgK
flgL	18255	19208	-	1-954/954	0/0	100.00	96.75	gi:15801200	flagellar hook-associated protein FlgL
flgN	29411	29827	+	1-417/417	0/0	100.00	98.80	gi:15801187	flagella synthesis protein FlgN
flhA	12066	14142	-	1-2077/2079	0/0	99.90	98.51	gi:15802291	flagellar biosynthesis protein FlhA
flhB	14135	15283	-	1-1149/1149	0/0	100.00	98.35	gi:15802292	flagellar biosynthesis protein FlhB
flhC	26358	26936	-	1-579/579	0/0	100.00	99.14	gi:15802303	transcriptional activator FlhC
flhD	26939	27298	-	1-360/360	0/0	100.00	99.72	gi:15802304	transcriptional activator FlhD
flhE	11672	12064	-	1-393/393	0/0	100.00	96.95	gi:15802290	flagellar protein
fliA	143	862	+	1-720/720	0/0	100.00	98.89	gi:15802357	flagellar biosynthesis sigma factor
fliE	8852	9166	-	1-315/315	0/0	100.00	97.14	gi:15802372	flagellar hook-basal body protein FliE
fliF	9381	11039	+	1-1659/1659	0/0	100.00	98.73	gi:15802373	flagellar MS-ring protein
fliG	11032	12027	+	1-996/996	0/0	100.00	99.10	gi:15802374	flagellar motor switch protein G
fliH	12020	12706	+	1-687/687	0/0	100.00	98.11	gi:161367588	flagellar assembly protein H

fliI	12706	14079	+	1-1374/1374	0/0	100.00	98.25	gi:161367587	flagellum-specific ATP synthase
fliJ	14098	14541	+	1-444/444	0/0	100.00	100.00	gi:15802377	flagellar biosynthesis chaperone
fliK	14538	15665	+	1-1128/1128	0/0	100.00	97.25	gi:15802378	flagellar hook-length control protein
fliL	15770	16234	+	1-465/465	0/0	100.00	98.71	gi:15802379	flagellar basal body-associated protein FliL
fliM	16239	17243	+	1-1005/1005	0/0	100.00	99.20	gi:15802380	flagellar motor switch protein FliM
fliN	17240	17653	+	1-414/414	0/0	100.00	98.79	gi:15802381	flagellar motor switch protein FliN
fliO	17656	18021	+	1-366/366	0/0	100.00	98.36	gi:161367586	flagellar biosynthesis protein FliO
fliP	18021	18758	+	1-738/738	0/0	100.00	97.70	gi:15802383	flagellar biosynthesis protein FliP
fliQ	18768	19037	+	1-270/270	0/0	100.00	97.41	gi:15802384	flagellar biosynthesis protein FliQ
fliR	19046	19831	+	1-786/786	0/0	100.00	97.20	gi:15802385	flagellar biosynthesis protein FliR
fliS	3289	3677	+	23-411/411	0/0	94.65	98.72	gi:15802360	flagellar protein FliS
fliT	3677	4042	+	1-366/366	0/0	100.00	97.54	gi:15802361	flagellar biosynthesis protein FliT
fliY	1547	2347	+	1-801/801	0/0	100.00	98.25	gi:15802355	cystine transporter subunit
fliZ	872	1459	+	1-588/588	0/0	100.00	98.13	gi:15802356	flagella biosynthesis protein FliZ
flk	19397	20392	+	1-996/996	0/0	100.00	98.69	gi:15802868	flagella biosynthesis regulator
gspK	9347	10324	-	1-978/978	0/0	100.00	98.26	gi:157155920	general secretion pathway protein K
gspL	8172	9350	-	1-1179/1179	0/0	100.00	99.75	gi:157156601	GspL-like protein
gspM	7634	8170	-	1-537/537	0/0	100.00	99.25	gi:157154814	putative general secretion pathway protein YghD
gspo	4021	4698	-	1-678/678	0/0	100.00	99.70	SPG000100	Prepilin peptidase Secretion system Type II Secretion System
hlyE	7622	8665	+	1-1056/1056	1/12	98.86	97.35	gi:209398688	hemolysin E
hofB	47348	48733	+	1-1386/1386	0/0	100.00	97.55	gi:15799791	hypothetical protein
hofC	48723	49925	+	1-1203/1203	0/0	100.00	97.51	gi:15799790	type IV pilin biogenesis protein
hofq	23225	24463	-	1-1239/1239	0/0	100.00	98.79	SPG000051	Outer membrane secretin Adherence Hemorrhagic coli pilus

ibeB	3082	4463	+	1-1382/1383	0/0	99.93	99.78	gi:443616608	copper/silver efflux system outer membrane protein CusC
ibeC	21320	23053	-	1-1734/1734	0/0	100.00	99.94	gi:386616783	hypothetical protein
motA	25344	26231	-	1-888/888	0/0	100.00	98.99	gi:15802302	flagellar motor protein MotA
motB	24421	25347	-	1-927/927	0/0	100.00	97.84	gi:15802301	flagellar motor protein MotB
nada	30012	31055	-	1-1044/1044	0/0	100.00	99.90	SPG000048	Escherichia coli str. K-12 substr. MG1655 complete genome
nadb	14097	15719	-	1-1623/1623	0/0	100.00	98.15	SPG000049	Escherichia coli str. K-12 substr. MG1655 complete genome
orgA	23344	23925	-	1-582/582	0/0	100.00	96.56	gi:15832969	hypothetical protein
orgB	22698	23129	-	1-432/432	0/0	100.00	95.83	gi:15832967	hypothetical protein
ppda	902	1372	-	1-471/471	0/0	100.00	98.73	SPG000131	Pilin-like protein minor pilin or pseudo pilin Adherence Hemorrhagic coli pilus
ppdb	348	911	-	1-564/564	0/0	100.00	98.58	SPG000112	Pilin-like protein minor pilin or pseudo pilin Adherence Hemorrhagic coli pilus
ppdc	14835	15158	-	1-324/324	0/0	100.00	98.77	SPG000140	Pilin-like protein minor pilin or pseudopilin Adherence Hemorrhagic coli pilus
ppdD	46898	47338	+	1-441/441	0/0	100.00	99.32	gi:15799792	putative major pilin subunit
stgA	27971	28513	-	31-573/573	0/0	94.76	99.82	gi:46487739	StgA
stgB	27192	27923	-	1-732/732	0/0	100.00	99.86	gi:46487740	StgB
stgC	24645	27167	-	1-2523/2523	0/0	100.00	97.58	gi:46487741	StgC
tar/cheM	20122	21783	-	1-1662/1662	0/0	100.00	98.31	gi:15802298	methyl-accepting chemotaxis protein II
ycbQ	71954	72502	+	1-549/549	0/0	100.00	99.27	gi:15800799	putative fimbrial-like protein
ycbR	72585	73286	+	1-702/702	0/0	100.00	97.44	gi:15800800	putative chaperone
ycbS	73311	75911	+	1-2601/2601	0/0	100.00	98.58	gi:15804976	PapC-like porin protein involved in fimbrial biogenesis
ycbU	787	1263	-	1-477/477	0/0	100.00	99.79	gi:15800802	hypothetical protein

ycbV	264	827	-	1-564/564	0/0	100.00	99.11	gi:15800803	putative fimbrial-like protein
ycfz	11173	11961	-	1-789/789	2/2	99.87	90.38	SPG000092	putative factor
ygdb	1	351	-	1-351/408	0/0	86.03	95.16	SPG000137	Pilin-like protein minor pilin or pseudo pilin Adherence Hemorrhagic coli pilus
ygeG	18662	19148	+	6-492/492	0/0	98.98	97.12	gi:15832962	hypothetical protein
ygeH	19483	20858	+	1-1377/1377	1/1	99.93	97.75	gi:15832963	transcriptional regulator
yggr	430	1410	+	1-981/981	0/0	100.00	97.86	SPG000074	Retraction ATPase Adherence Hemorrhagic coli pilus
Z1307	59269	60309	+	1-1041/1041	0/0	100.00	99.81	gi:15800816	outer membrane protein A
Z2206	15158	16072	-	1-915/915	0/0	100.00	97.60	gi:15801636	putative adhesin; FimH protein
