# Supplementary material for: Whole-Genome Sequencing of Escherichia coli O18ab:H11 from South African Beef: Antimicrobial Resistance, Virulence, and a Rare Sequence Type
Source: Microorganisms. 2026 Sep 9;14(9):2003. doi: 10.3390/microorganisms14092003 (PMC13609402; doi:10.3390/microorganisms14092003)
Supplement: Supplementary file 1 [file microorganisms-14-02003-s001.zip › Table S4.pdf]

Table S4: Virulence gene categories and key functions identified in the beef-derived *E. coli* isolate (EM-5_S15).

Category	Genes observed	Key function
**Curli (biofilm)**	*csgA–G*	Amyloid fibers for biofilm & adhesion
**Type 1 fimbriae**	*fimA–I*	Mannose-sensitive adhesion; *FimH* tip adhesin
***E. coli* common pilus**	ecpRABCDE	Epithelial adherence; UPEC colonization support
**Motility / Chemotaxis**	*flgA-L/N, flhA-E, fliA/E-T/Y/Z, flk, cheA/B/R/W/Y/Z, tar*	Flagellum assembly & chemotaxis regulation
**Iron acquisition**	*entA–F/S, fepA–G, fes*	Enterobactin siderophore system
**Toxin**	*hlyE (ClyA)*	Pore-forming cytolysin
**Invasion**	*ibeB, ibeC*	Brain endothelial invasion in NMEC models
**T3SS-related (non-LEE)**	*espX1/4/5, espL1/3/4*	Non-LEE effectors in A/E pathogens (not pathotype-defining alone)
