# Supplementary material for: Whole-Genome Sequencing of Escherichia coli O18ab:H11 from South African Beef: Antimicrobial Resistance, Virulence, and a Rare Sequence Type
Source: Microorganisms. 2026 Sep 9;14(9):2003. doi: 10.3390/microorganisms14092003 (PMC13609402; doi:10.3390/microorganisms14092003)
Supplement: Supplementary file 1 [file microorganisms-14-02003-s001.zip › Table S8.pdf]

Table S8: Distribution of the 398 African *E. coli* genomes by country and isolation source/type.

Country	Clinical	Human carriage	Human fecal	Meat	Chicken	Waste/animal	Research	Not recorded	Total
Niger (NE)	0	177	0	0	0	0	0	0	177
Tanzania (TZ)	48	0	0	0	0	0	0	0	48
Ethiopia (ET)	6	0	31	1	5	4	0	0	47
Egypt (EGY)	34	0	0	0	0	0	0	0	34
Tunisia (TN)	0	0	0	33	0	0	0	0	33
Mali (ML)	24	0	0	0	0	0	0	0	24
Zambia (ZM)	14	0	0	0	0	0	0	0	14
Ghana (GH)	8	0	0	0	0	0	0	0	8
Kenya (KE)	2	3	0	0	0	0	0	0	5
South Africa (ZA)	0	0	0	1	0	0	0	2	3
Nigeria (NG)	0	0	0	0	0	0	3	0	3
Algeria (DZ)	2	0	0	0	0	0	0	0	2
Total	138	180	31	35	5	4	3	2	398
